# Detection of internal N7-methylguanosine (m^7^G) RNA modifications by mutational profiling sequencing

**DOI:** 10.1093/nar/gkz736

**Published:** 2019-08-31

**Authors:** Christel Enroth, Line Dahl Poulsen, Søren Iversen, Finn Kirpekar, Anders Albrechtsen, Jeppe Vinther

**Affiliations:** 1 Department of Biology, University of Copenhagen, Ole Maaløes Vej 5, DK-2200 Copenhagen N, Denmark; 2 Department of Biochemistry and Molecular Biology, University of Southern Denmark, Campusvej 55, DK-5230 Odense M, Denmark

## Abstract

Methylation of guanosine on position N7 (m^7^G) on internal RNA positions has been found in all domains of life and have been implicated in human disease. Here, we present m^7^G Mutational Profiling sequencing (m^7^G-MaP-seq), which allows high throughput detection of m^7^G modifications at nucleotide resolution. In our method, m^7^G modified positions are converted to abasic sites by reduction with sodium borohydride, directly recorded as cDNA mutations through reverse transcription and sequenced. We detect positions with increased mutation rates in the reduced and control samples taking the possibility of sequencing/alignment error into account and use replicates to calculate statistical significance based on log likelihood ratio tests. We show that m^7^G-MaP-seq efficiently detects known m^7^G modifications in rRNA with mutational rates up to 25% and we map a previously uncharacterised evolutionarily conserved rRNA modification at position 1581 in *Arabidopsis thaliana* SSU rRNA. Furthermore, we identify m^7^G modifications in budding yeast, human and arabidopsis tRNAs and demonstrate that m^7^G modification occurs before tRNA splicing. We do not find any evidence for internal m^7^G modifications being present in other small RNA, such as miRNA, snoRNA and sRNA, including human Let-7e. Likewise, high sequencing depth m^7^G-MaP-seq analysis of mRNA from *E. coli* or yeast cells did not identify any internal m^7^G modifications.

## INTRODUCTION

It is becoming increasingly clear that RNA modifications play important roles in many cellular processes and evidence linking the proteins responsible for modification of RNA to the development of human diseases is accumulating (1). This is also true for methylation of guanosine position 7 (m^7^G), which is present at internal positions in both tRNA and rRNA. In the yeast small ribosomal subunit (SSU) rRNA, position 1575 is m^7^G modified by the Bud23-Trm112 heterodimer (2,3) and this modification is conserved to human SSU rRNA position 1639 (4,5). Most bacterial ribosomes are also m^7^G modified, including the *Escherichia coli* ribosome, which has a m^7^G modification both on the SSU and the LSU rRNAs (6,7). Likewise, some tRNAs has a conserved m^7^G modification at position 46, which stabilises the tertiary tRNA fold by improving the geometry of a base triple N13-N22-m^7^G46 (8,9). The set of tRNAs carrying the m^7^G modification is only known with some confidence in yeast (10,11) and recently also in mice (12) and humans (13).

The mapping of RNA modifications has been improved by the development of a variety of sequencing based global detection methods, which in some cases has allowed mRNA modifications to be detected (14). The detection of the N^1^-methyladenosine (m^1^A) RNA modification is an example of this development. The m^1^A modification causes stops or misincorporations during reverse transcription. Initially, m^1^A was detected on a global scale using specific antibodies to immunoprecipitate m^1^A modified RNA combined with detection of the reverse transcriptase stop events resulting from m^1^A (15,16). Subsequently, the availability of thermostable group II intron reverse transcriptase fusion proteins (TGIRT) made it possible to increase the proportion of m^1^A reverse transcriptase read-throughs compared to stops. This allowed m^1^A immunoprecipitation to be combined with mutational profiling of m^1^A, thereby increasing the signal to noise ratio and improving m1A detection (17–19). RNA modifications, including m^1^A, are abundant in tRNA and their interference with reverse transcription makes mapping of tRNA modifications challenging. Treatment with the AlkB demethylase and use of TGIRT for reverse transcription therefore improves tRNA profiling and allows global detection of m^1^A in tRNA (20).

The m^7^G modification on the Hoogsteen edge does not interfere with reverse transcription, but is specifically sensitive to mild reduction with sodium borohydride (NaBH_4_), which has been exploited to create abasic sites at m^7^G modified RNA positions (21,22). After subsequent treatment with aniline, the RNA will be cleaved at the abasic sites, thereby allowing detection by mapping of reverse transcription stops (11,21,22). This strategy forms the basis for the tRNA reduction and cleavage sequencing (TRAC-Seq method), which were used together with an m^7^G specific antibody based method (m^7^G MeRIP-seq) to confidently map the m^7^G modifications on mouse tRNAs (12). More recently and while this manuscript was in review, two additional papers describing m^7^G mapping methods were published (13,23). Both of the studies combined m^7^G MeRIP-seq results with results from methods based on RNA reduction with NaBH_4_ and biotinylation of abasic sites with biotin reagents. Pandolfini *et al.* developed a method called Borohydride Reduction sequencing (BoRed-seq) based on pull-down of the biotinylated RNA fragments followed by sequencing and applied it to human miRNAs, which led to the discovery of several human miRNAs with m^7^G modifications, including Let-7e, which was further characterized (23). Zhang *et al.* detected m^7^G modifications at nucleotide resolution by mapping of mutations occurring as a result of misincorporation at the biotinylated sites during reverse transcription and use their methods to map 803 m7G mRNA modifications in human mRNA as well as m^7^G modifications in human tRNAs (13).

Here, we describe the m^7^G Mutational Profiling sequencing (m^7^G-MaP-seq) method for high throughput nucleotide resolution mapping of internal m^7^G modifications. Our method is based on NaBH_4_ treatment of RNA and direct detection of mutations resulting from misincorporation at abasic sites during reverse transcription. We show that m^7^G-MaP-seq detects known and novel m^7^G modifications in rRNA and tRNA with mutational rates of up to 25%. We find that arabidopsis SSU rRNA has an m^7^G modification at position 1581, which corresponds to the location of the SSU rRNA position modified both in human and yeast rRNA, suggesting that the SSU m^7^G modification is universally conserved in eukaryotes. Moreover, by applying m^7^G-MaP-seq to small RNA sequencing libraries, we detect tRNA m^7^G modifications from yeast, human and Arabidopsis. In contrast, m^7^G-MaP-seq analysis of other types of short RNAs and of *E. coli* and yeast mRNAs failed to identify any internally m^7^G modified positions.

## MATERIALS AND METHODS

### Cell culture and RNA isolation

Yeast *MATa* strain BY4741 (cat. no. YSC1048) were obtained from GE Healthcare Dharmacon (Dharmacon Yeast Knockout collection). The yeast cells were grown at 30°C in YPD medium. At OD = 0.75, the cells were collected by centrifugation, and pellets were washed with ice-cold water and re-suspended in 400 μl ice-cold TES solution (10 mM Tris–HCl pH 7.5, 10 mM EDTA pH 8.0, 0.5% SDS). RNA was extracted using a hot acid phenol protocol.

The *E. coli* Keio Knockout RsmG strain (OEC4987-213607518) and the parental BW25113 strain (OEC5042) were obtained from GE Healthcare Dharmacon (*E. coli* Keio Knockout panel). The strains were inoculated in Luria-Bertani (LB) media (containing 50 μg/ml kanamycin for the knockout strains). Overnight cultures were diluted 1:100 and at OD_600_ = 0.4, ice-cold EtOH/Phenol stop solution (5% saturated phenol (pH < 7.0) 95% ethanol) was added (6.25 μl per 25 ml cell culture). Cells from 25 ml culture were pelleted at 3000 g for 5 minutes at 4°C and resuspended in 800 μl TE buffer (100 mM Tris, 10 mM EDTA, pH. 8.0) with 0.5 mg/ml lysozyme, followed by the addition of 1/10 vol of 10% Sodium dodecyl sulphate (SDS). After incubation at 64°C for 2 min, 1/10 vol of 1 M NaOAc, pH 5.2 was added, followed by addition of an equal volume of water saturated phenol (pH < 7.0). After incubation at 64°C for 6 min and centrifugation at 14 000 g for 10 min at 4°C, the aqueous layer was transferred to a new tube containing an equal volume of chloroform, inverted repeatedly, and centrifuged at 14 000 g for 5 min at 4°C. The aqueous layer was ethanol precipitated, and the RNA pellet was dissolved in RNase-free water.

*Arabidopsis* Columbia-0 seeds were washed in 96% ethanol followed by 10% bleach, spread onto solid agar plates and incubated 48 h at 4°C in darkness. Next, the seeds were incubated under light and harvested at day 14. The plant tissue was grinded in liquid nitrogen, and 100 mg of the grinded tissue was mixed with 1 ml of Trizol (Invitrogen, cat # 15596026), followed by addition of 200 μl chloroform. After vortexing, samples were centrifuged at 14 000 g at 4°C for 10 minutes and 500 μl of the supernatant was mixed with 500 μl isopropanol and incubated at room temperature for 30 minutes. RNA was pelleted by centrifugation at 14 000 g at 4°C for 15 min and washed with twice with 70% ethanol before resuspension in RNase-free water.

HeLa cells were cultured in DMEM medium containing 10% Foetal Bovine Serum, 1% l-glutamate, 1% penicillin/streptomycin. The cells were harvested at ∼80% confluency and RNA was isolated using Trizol reagent (Invitrogen, cat. # 15596026) according to the manufacturer's protocol.

### DNase treatment

DNA was depleted from the RNA using the RNase Free DNase Set (Qiagen, cat. # 79254) according to manufacturer's protocol. The RNA quality was assessed on an Agilent 2100 Bioanalyzer using a RNA pico assay (RIN values were >9).

### mRNA enrichment

RNA from yeast were poly(A)-enriched, using the Poly(A) Purist MAG Kit (Ambion, cat. # AM1922), using the standard protocol. The enrichment procedure was performed twice. RNA from *E. coli* was rRNA depleted using the Ribo Zero rRNA removal Kit (Illumina, cat. # MRZGN126). Poly-A enriched or rRNA depleted RNA was purified with Agencourt Ampure XP RNAclean beads (Beckman Coulter, cat. # A63987) in a sample:beads ratio of 1:1.2, to reduce the small RNA fraction. The purification was performed according to the manufacturer's protocol and the purified RNA was eluted in 10 μl RNase-free water.

### Fragmentation

The RNA was mixed with an equal volume of 2× fragmentation buffer (100 mM Tris–HCl (pH 8), 10 mM MgCl_2_), and the samples were incubated at 95°C for 3 min and 20 s. The samples were then placed on ice and EDTA was added to a final concentration of 10 mM. The RNA was purified with Agencourt Ampure XP RNAclean beads (Beckman Coulter) in a sample:beads ratio of 1:3.6, and the purified RNA was eluted in 10 μl of 1 M Tris pH 8.2.

### NaBH_4_ reduction

Fragmented RNA was either incubated with 0.3 M freshly prepared NaBH_4_ (NaBH_4_ (+) samples) or with RNase-free water (NaBH_4_ (–) samples). The pH of the 0.3 M NaBH_4_ solution was 9. The reactions were incubated on ice for 30 min in darkness, followed by ethanol precipitation of the RNA, which were used directly for reverse transcription to preserve abasic sites.

### First-strand cDNA synthesis (total RNA and mRNA samples)

Reverse transcription was performed with the PrimeScript Reverse Transcriptase (Takara Bio) in 35 μl reactions using 10 μl of RNA as template. 100 μM reverse transcription primer (RT_random_primer, see supplementary S1) was added to RNA sample and the mixture was incubated at 65°C for 5 minutes and placed immediately on ice. Next 7.5 μl 5× Primescript buffer, 7.5 μl 3.3 M/0.6 M sorbitol–trehalose mix, 1.9 μl 10 mM dNTP, 7.6 μl water and 1.5 μl Primescript reverse transcriptase to each reaction and reverse transcription was carried out at 25°C for 30 s, 30°C for 10 min, 42°C for 30 min, 50°C for 10 min, 56°C for 10 min, 60°C for 10 min. The cDNA was purified with Agencourt Ampure XP beads (Beckman Coulter) in a sample:beads ratio of 1:1.8, and eluted in 35 μl water. The cDNA was then vacuum concentrated to 7 μl.

### 3′-end adapter ligation

Ligation of the adapter to the 3′-end of the cDNA was performed using CircLigase ssDNA Ligase (Epicentre) in 10 μl reactions. Initially, 3 μl RNA/cDNA duplex were mixed with 0.5 μl 100 μM Ligation_adapter (see supplementary S1) on ice, followed by addition of 6.5 μl master mix consisting of 1 μl CircLigase buffer, 0.5 μl ATP, 0.5 μl MnCl_2_, 2 μl 50% PEG 6000, 2 μl betaine and 0.5 μl CircLigase enzyme. Reactions were transferred to a thermocycler and incubated 2 h at 60°C, 1 h at 68°C, and 10 min at 80°C to inactivate the enzyme. The cDNA was purified with Agencourt Ampure XP beads (Beckman Coulter) in a sample:beads ratio of 1:1.8, and the purified cDNA was eluted in 10 μl water.

### Library amplification by PCR

The amplification of the library was conducted using Phusion^®^ High-Fidelity DNA Polymerase (New England Biolabs, Cat #m0530) and custom Illumina-compatible primers (see supplementary S1). Reactions of 50 μl were prepared and consisted of 7 μl single stranded cDNA, 3 μl forward primer, 2.5 μl reverse index primer, 10 μl Phusion 5× HF buffer, 4 μl 2.5 mM dNTPs, 22.5 μl water and 1 μl Phusion Polymerase.

The reactions were denatured for 3 min at 98°C, followed by 4 cycles of 98°C for 80 s, 64°C for 15 s and 72°C for 1 min followed by 14–17 cycles of 98°C for 80 s and 72°C for 1 min and subsequently 72°C for 5 min.

The samples were pooled and run on the E-Gel^®^ iBase™ Power System (Thermo Fisher) with the ‘SizeSelect 2%’ program to remove primer-adapter contamination. Following collection of the 150–600 bp fraction, the pooled library was ethanol precipitated, and sequenced on the Illumina NextSeq platform.

### Small RNA library preparation

Total RNA (1 μg) with a RIN value of 9.8 or higher was either NaBH_4_ or mock treated as described in the ‘NaBH_4_ reduction’ section. Small RNA libraries of yeast, human and arabidopsis were prepared using ‘The NEBNext Multiplex Small RNA’ kit according to manufacturer's protocol. The pooled libraries were run on the E-Gel^®^ iBase™ Power System (Thermo Fisher) with the ‘SizeSelect 2%’ program, to remove 5.8s rRNA and to selectively exclude the >250 bp fraction. The pooled libraries were ethanol precipitated and sequenced on the Illumina NextSeq platform.

### SSU rRNA sub-fragment isolation and mass spectrometry analysis

An ∼50 nucleotide fragment of the *A. thaliana* SSU rRNA sequence around G1581 was isolated for MALDI Time-of-Flight mass spectrometric analysis. Purified total RNA was hybridized with a complementary oligodeoxynucleotide Ara_LSU_1581 (Supplementary Table S1), followed by digestion with mung bean nuclease and RNase A as described in (24). After sub-fragment purification on a denaturing polyacrylamide gel, the RNA was separately digested with RNase A (pyrimidine-specific) for mass spectrometric analysis as previously reported (25). Briefly, 1–2 pmol rRNA sub-fragment were RNase A digested to completion and analysed using 3-hydroxypicolinic acid as matrix. Mass spectra were recorded in positive ion mode with a reflectron Time-of-Flight mass analyzer on a Bruker UltraFlex MALDI instrument and processed using the software ‘*m*/*z*’ (Proteometrics). The observed digestion pattern was compared to a theoretical *ditto* calculated using GPMAW software (Lighthouse Data, Denmark). Tandem mass spectrometry to investigate exact positions of the observed modifications was performed on a Waters Q-TOF Premiere instrument (Waters, Manchester, UK) equipped with a MALDI ion source in positive ion mode as previously described (26).

### Reads pre-processing and mapping

All raw data files and the fines processed datafiles containing the data used for making figures are available at GEO under accession GSE121927. The analysis is described in detail at https://github.com/jeppevinther/m7g_map_seq. In brief, sequencing reads from regular RNA-Seq experiments were processed with Cutadapt to remove adapter sequences and trim dark cycle artefacts from the two colour Nextseq platform using the options -a AGATCGGAAGAGCACACGTCT –nextseq-trim = 20’. The preprocessing.sh script from RNAProbR package (27) (http://people.binf.ku.dk/∼jvinther/data/rna_probing/) was used to remove the 7 base barcode from the sequencing read using the options ‘-b NNNNNNN -t 9’ and the barcode information was saved in a separate file. Reads having one or more Ns in the barcode sequence were discarded before mapping with bowtie2 using high sensitivity options ‘–local -N 1 -D 20 -R 3 -L 15’.

For analysis of ribosomal rRNA, reads were mapped to ribosomal sequences obtained from the CRW database (28). For E. coli mRNA analysis, reads were mapped to the ensembl cDNA release 87 for the bw25113 strain ftp://ftp.ensemblgenomes.org/pub/release-29/bacteria//fasta/bacteria_87_collection/escherichia_coli_bw25113/cdna/Escherichia_coli_bw25113.GCA_000750555.1.29.cdna.all.fa.gz. For yeast mRNA analysis, reads were mapped to the ensembl cDNA release 84 of S. cerevisiae ftp://ftp.ensembl.org/pub/release-84/fasta/saccharomyces_cerevisiae/cdna/Saccharomyces_cerevisiae.R64-1-1.cdna.all.fa.gz. Potential PCR duplicates among the sequencing reads were removed by discarding all, but one of the reads having identical barcode and mapping to the same position using the collapse.sh script (http://people.binf.ku.dk/jvinther/data/RNA-seq).

Sequencing reads from small RNA-Seq experiments were processed and mapped using the settings described above but as these reads do not include the adapter barcode, no pre-processing and collapsing of PCR duplicates on barcodes were performed. For tRNA analysis of yeast, human and arabidopsis, reads were mapped to the high confidence tRNA fasta sequences obtained from the genomic tRNA database (http://gtrnadb.ucsc.edu/index.html). Prior to mapping, redundant/identical RNA sequences from different genes were collapsed, tRNA introns were removed from the sequences and 3′ terminal CCA were added. For histidine tRNAs a 5′ G were added to the sequence. In the analysis of unspliced tRNA, both the spliced and unspliced tRNA sequences were included in the fasta sequence used for mapping. For the analysis of Arabidopsis and human miRNAs, reads were mapped to the miRbase miRNA precursor annotation ftp://mirbase.org/pub/mirbase/CURRENT/hairpin.fa.gz. Sequences for other sRNA were obtained using BioMart from the Ensembl human gene (GRCh38.p12) annotation with a filter restricting to genes annotated as ‘scaRNA, scRNA, snoRNA, snRNA, sRNA’ and Arabidopsis thaliana gene (TAIR10) annotation with a filter restricting to genes annotated as ‘snoRNA, snRNA, SRP_RNA’.

### Detection of mutations and statistical analysis

The mapped reads for the control and NaBH_4_ treated samples were processed with the mpileup tool from the samtools package using per-Base Alignment Quality, which estimates the probability of a read base being wrongly aligned (29,30). To parse the mpileup output into tabular form and perform statistical analysis, we developed an R-script (getFreq2000.R) based on the statistical principles developed for the ANGSD package (31). The statistics employed in the script is described in the Supplementary methods and the getFreq2000.R script is available at https://github.com/jeppevinther/m7g_map_seq. Briefly, the R function takes a samtools mpileup output file as input and outputs a number of statistics for each position, including the sequencing depth, the estimated mutation frequency for the treated and control samples taking the probability of a read base being wrongly aligned into account. The mutation rate difference used for plotting in most of our figures is the estimated mutation frequency for the control subtracted from estimated mutation frequency for the treated sample. In addition, the script uses log likelihood tests to calculate *P*-values for the estimated mutation rates being different in the treated and the control samples. Likewise, it also tests for difference mutation rates within the replicate samples. For a full description of the output of the R script, see Supplementary Table S2.

### Data analysis

The output from the getFreq function was used for plotting and further data analysis in R. For the rRNA analysis, only positions having a sequencing depth higher than 500 was used. For the tRNA and mRNA analysis, only positions having a combined sequencing depth higher than 1500 in the six samples, an average mutation frequency less than 1% in the control replicates, and no significant difference in mutation rates (*P*-value < 10^−5^) within control or NaBH_4_ treated replicates were used for preparing figures. The Modomics database (10) was used for annotation of tRNA m^7^G, m^1^A and dihydrouridine positions.

## RESULTS

### m^7^G-MaP-seq detects m^7^G modifications

**Figure 1.**
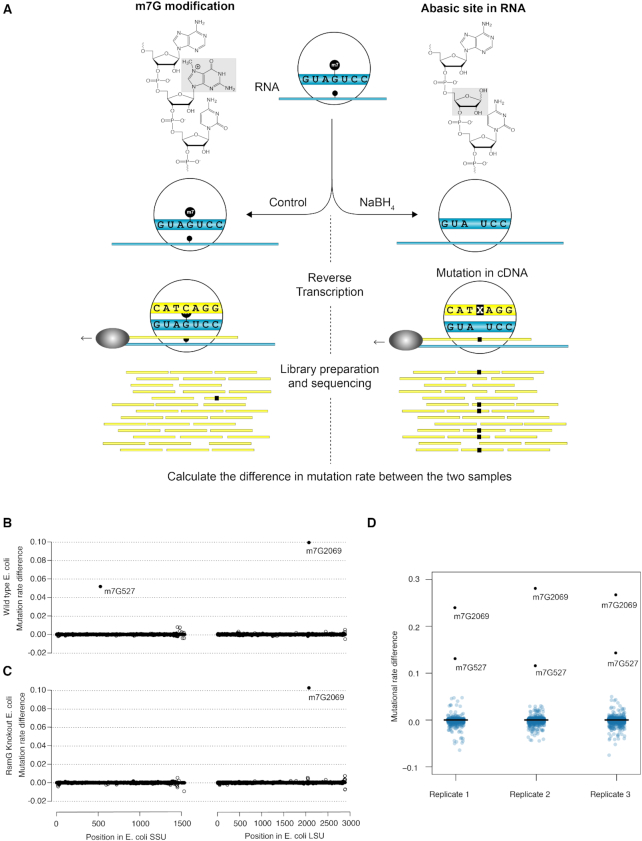
(**A**) Schematic representation of the m7G-MaP-Seq method. NaBH4 treatment leads to formation of abasic sites at m7G positions, which subsequently are converted into cDNA mutations during reverse transcription and detecte by massive parallel sequencing. (**B**) Detection of m7G at E. coli SSU rRNA position 527 and E. coli LSU rRNA position 2069 in WT strain. (**C**) Detection of m7G at E. coli SSU rRNA position 527 and E. coli LSU rRNA position 2069 in RsmG knockout strain. (**D**) Boxplots showing consistent detection of m7G modified positions in biological replicates.

In m^7^G-MaP-seq, we exploit the specific sensitivity of m^7^G to mild reduction with sodium borohydride (NaBH_4_), which lead to formation of RNA abasic sites (Figure 1A). In contrast to previous methods, we preserve the abasic sites in the RNA and use reverse transcription with the Primescript MMLV reverse transcriptase to record the positions of m^7^G modifications directly as mutations in the cDNA primary sequence (Figure 1A and Supplementary Figure S1). The cDNA is used to prepare libraries for massive parallel sequencing and the resulting data is mapped to a relevant reference using local alignments and high sensitivity settings. To perform data analysis and detect positions with increased mutation rates in the reduced samples compared to control samples, we developed a pipeline based on the mpileup tool from the samtools package (29,30) and a new tool called getFreq, which we developed based on the ANGSD package (31). Our new tool takes the observed sequenced bases and the possibility of sequencing/alignment error into account to calculate estimated mutation rates for the treated and control samples, which are subtracted to give a mutation rate difference for each position. Moreover, our tool uses replicates samples to calculate the statistical significance of the mutation rate difference based on log likelihood ratio tests and several other statistics (See Supplementary Table S2 and the Supplementary Methods). To validate the method, we first applied m^7^G-MaP-Seq to *E. coli* rRNA, where two m^7^G modifications are known. One is located in the conserved 530 loop of hairpin 18 in the bacterial SSU rRNA (m^7^G527 in *E. coli*) (7) and the other at position 2069 within the peptidyl transferase center of LSU rRNA (6). NaBH_4_ treatment specifically induced deletions and misincorporations at the two known m^7^G modified positions and resulted in mutation rate differences of 10% and 5% (Figure 1B), respectively. To demonstrate that our method specifically detects the m^7^G modifications and not some other feature of positions 527 and 2069, we repeated the experiment using a knockout strain for the RsmG methyltransferase known to carry out the modification at position 527 (7). As expected, we found that the knockout specifically eliminated NaBH_4_ treatment induced mutations at position 527, while the normalised mutation rate of any other position remained unaffected (Figure 1C). Next, we applied m^7^G-MaP-Seq on three biological replicates and found that the observed mutation rate differences for each position were highly reproducible between the replicates within the experiment (Figure 1D), whereas we observe some variation in the mutation rates obtained between different experiments (compare Figure 1B with 1D). To analyse the relationship between the modification frequency and the observed mutation rates, we first established that the *E. coli* SSU position 527 100% m7G modified in the WT stain and absent in the RsmG knock out strain using mass spectroscopy (Supplementary Figure S2A). We then prepared known mixtures of RNA isolated from the WT and RsmG strains and applied m^7^G-MaP-Seq to the samples. We find that there is a linear relationship between the known m^7^G modification frequency in the samples and the observed mutation rate differences, although were observed mutation rates were somewhat lower in this particular experiment (Supplementary Figure S2B). As expected for reverse transcription of abasic sites, we find that the m^7^G-MaP-Seq signal is composed of all possible types of mutations, including insertions and deletions (Supplementary Figure S2C). Both the observed mutation rates and types of mutations vary among different m^7^G positions, indicating that the flanking sequences influence the reverse transcription of abasic sites. Apart from mutations, we find that reverse transcription of the abasic sites leads to a relatively small relative increase in the count of reverse transcription (RT)-stops observed at the m^7^G modified positions (Supplementary Figure S3), indicating that the Primescript reverse efficiency reverse transcribes abasic sites at the reaction conditions used in m^7^G-MaP-Seq.

### m^7^G-MaP-seq detects known and novel m^7^G modifications in rRNA

**Figure 2.**
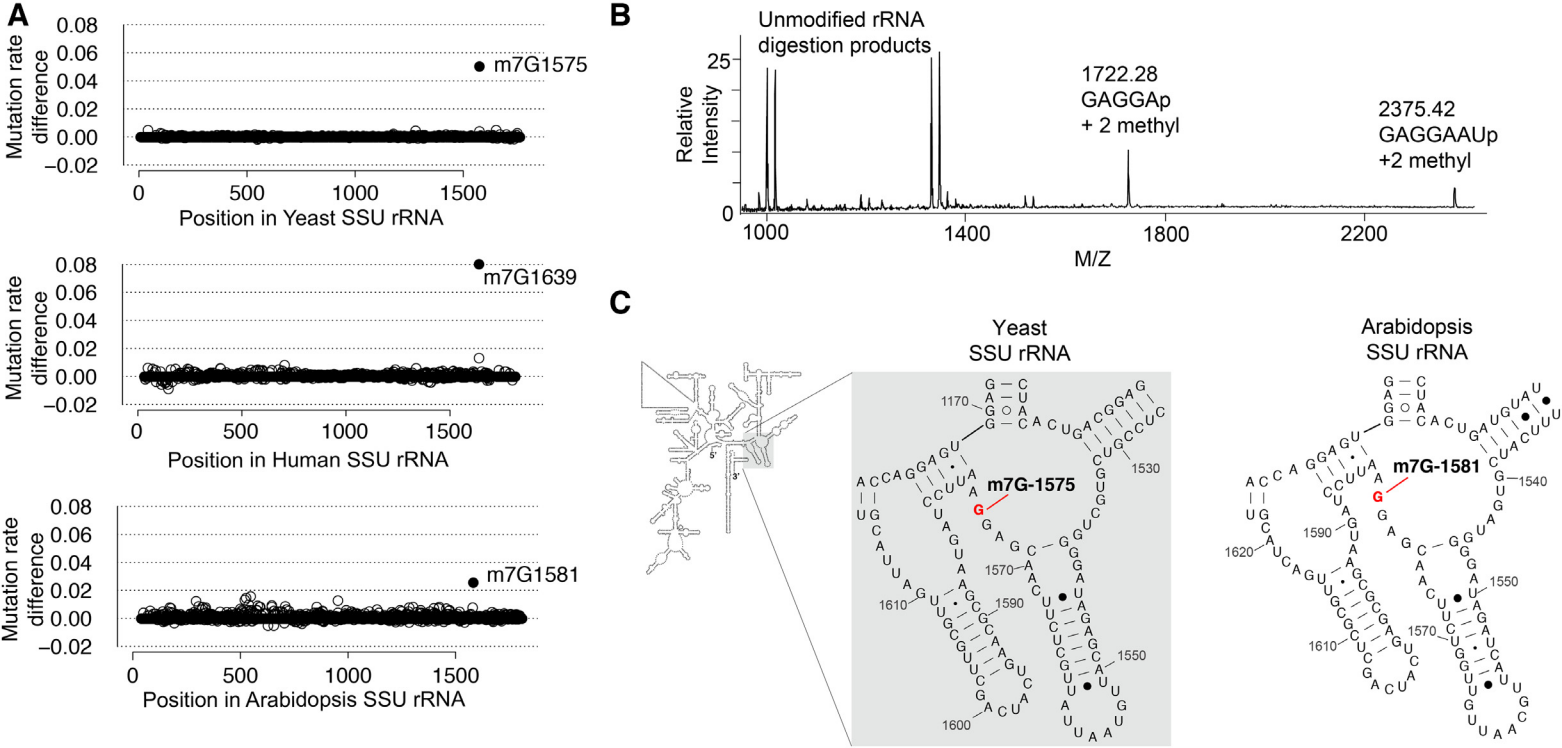
(**A**) Detection of m7G modifications in yeast, human and arabidopsis SSU rRNA. (**B**) Detection and validation of the m7G modification at Arabidopsis SSU rRNA position 1581 using mass spectroscopy. (**C**) Conservation of SSU rRNA m7G modification between yeast and Arabidopsis (secondary structures from the CRW database).

Next, we applied m^7^G-MaP-seq to total RNA isolated from yeast (*S. cerevisiae*), arabidopsis (*A. thaliana*) and human (*H. sapiens*) cells in a single replicate. For both human and yeast rRNA, we detect the known small subunit (LSU) rRNA m^7^G modification at positions G1639 and G1575, respectively (Figure 2). In arabidopsis SSU rRNA, no m^7^G methylation equivalent to the G1575 methylation in yeast has been described, but m^7^G-MaP-seq detects an m^7^G modification at position G1581, which aligns to the yeast SSU rRNA G1575 (Figure 2C). To further validate the arabidopsis m^7^G methylation at SSU G1581, we purified an RNA fragment surrounding the position and subjected it to RNase A digestion and Matrix-Assisted Laser Desorption/Ionization mass spectrometry (MALDI-MS) and tandem mass spectrometry. The mass spectrometry analysis demonstrates two methylations in the RNase A product G1578-U1584, m/z 2375.42—and a spontaneous truncation of this, G1578-A1582, *m*/*z* 1722.28—close to 100% modified (Figure 2B). Tandem mass spectrometry was applied to pinpoint the methylation in G1578-U1584 (Supplementary Figure S4) and G1578-A1582 (data not shown), where the two methyl groups were assigned to A1579 and G1581, respectively. The loss of a methylated G base (*m*/*z* 2210.35) shows that the methylation of position 1581 is on the guanine. This demonstrates that the SSU G1581 methylation is conserved in arabidopsis (Figure 2C).

### m^7^G-MaP-seq maps m^7^G modifications in tRNA

**Figure 3.**
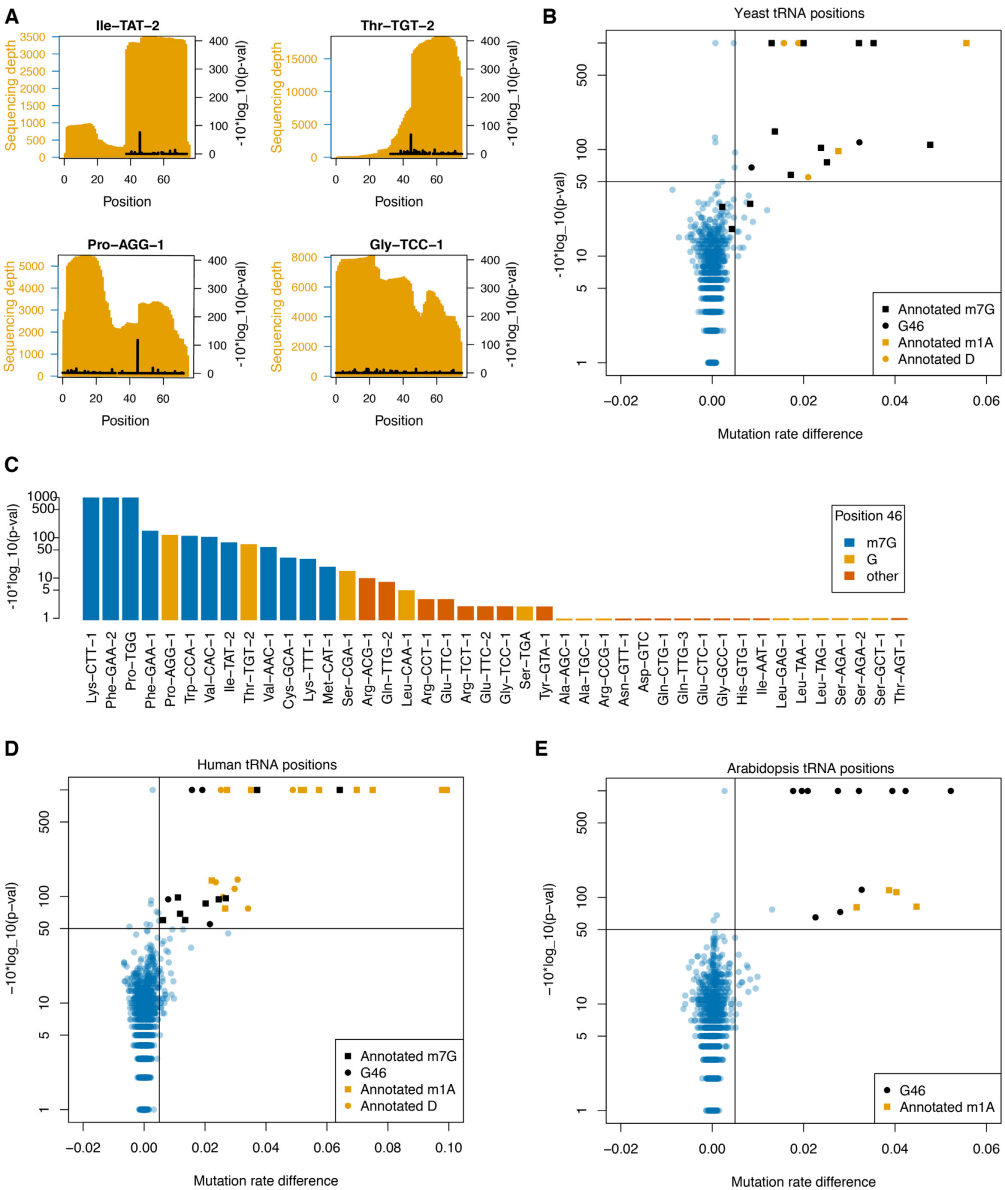
(**A**) Sequencing depth and m7G-MaP-seq -10*log_10(p-val) for selected yeast tRNA. (**B**) Mutation rate difference versus -10*log_10(*P*-val) plot of all detected yeast tRNA positions. Known m7G positions are specifically detected by m7G-MaP-seq. (**C**) m7G-MaP-seq -10*log_10(p-val) for yeast tRNA positions aligning to position 46 in the tRNA variable loop. (**D**) Mutation rate difference vs -10*log(p-val) plot of all detected human tRNA positions. (**E**) Mutation rate difference vs -10*log_10(p-val) plot of all detected arabidopsis tRNA positions.

A subset of tRNAs are m^7^G modified at position 46 in the variable loop and for yeast tRNAs, the m^7^G modifications have been annotated (10). We applied m^7^G-MaP-seq to small RNA isolated from yeast in biological triplicates with one treated and one control library per replicate. For tRNAs, we mapped to sequence files containing all known tRNA isodecoders and used high sensitivity mapping settings, which allows distinction of tRNA isodecoders differing in as little as one sequence position. When sequencing reads cover both the potential m^7^G modification at position 46 and the positions that differ between isodecoders, our analysis has the potential to detect isodecoder specific m^7^G modifications. The analysis was limited to tRNA positions with a sequencing depth of more than 1500 in the six samples combined, an average mutation frequency <1% in the control replicates, and no significant difference in mutation rates within replicates (see Methods for details). Some tRNAs, such as Ile-TAT-2, Pro-AGG-1 and Thr-TGT-2 showed highly significant mutation rate differences specifically at position 46, whereas others, such as tRNA-Gly-TCC-1 did not (Figure 3A).

To detect m^7^G modification on a global scale, we plotted the observed difference in mutation rate versus the *P*-value obtained for the comparison of the control samples with the NaBH_4_ treated samples for all positions (Figure 3B). Using a cut-off of 0.5% for the mutation rate difference and *P*-value threshold 10^−50^ for the difference between rates, we specifically predict all but three of the m^7^Gs annotated in the Modomics database for yeast tRNAs as having m^7^G modifications (Figure 3B) (10). The three tRNA for which no m^7^G modification is detected are Met-CAU, Lys-UUU and Cys-GCA and they all have increased relative frequency and a *P*-value <10^−10^ but does not reach our strict cut-off (Figure 3B). For two tRNAs (tRNA-Pro-AGG and tRNA-Thr-TGT) not previously known to be m^7^G modified, the m^7^G-MaP-seq data predict that position G46 is indeed modified (Figure 3C, Supplementary Table S3). Moreover, the m^7^G-MaP-seq analysis also shows that several tRNAs with G in position 46 do not show any signs of modification (Ala-AGC-1, Ala-TGC-1, Leu-GAG-1, Leu-TAA-1, Leu-TAG-1, Ser-AGA-1 and Ser-AGA-2, Ser-GCT-1).

In addition to m^7^G positions, some positions known to be N^1^-methyladenosine (m^1^A) and Dihydrouridine (D) modified also pass our filter and have increased relative mutation frequency and low *P*-values (Figure 3B). Dihydrouridine positions are known to be susceptible to NaBH_4_ reduction, potentially leading to the formation of abasic sites (32). For known m^1^A positions, we consistently find increased mutation rates in the NaBH_4_ treated samples, suggesting that m^1^A is susceptible to mild reduction with NaBH_4_ (Supplementary Figure S5).

**Figure 4.**
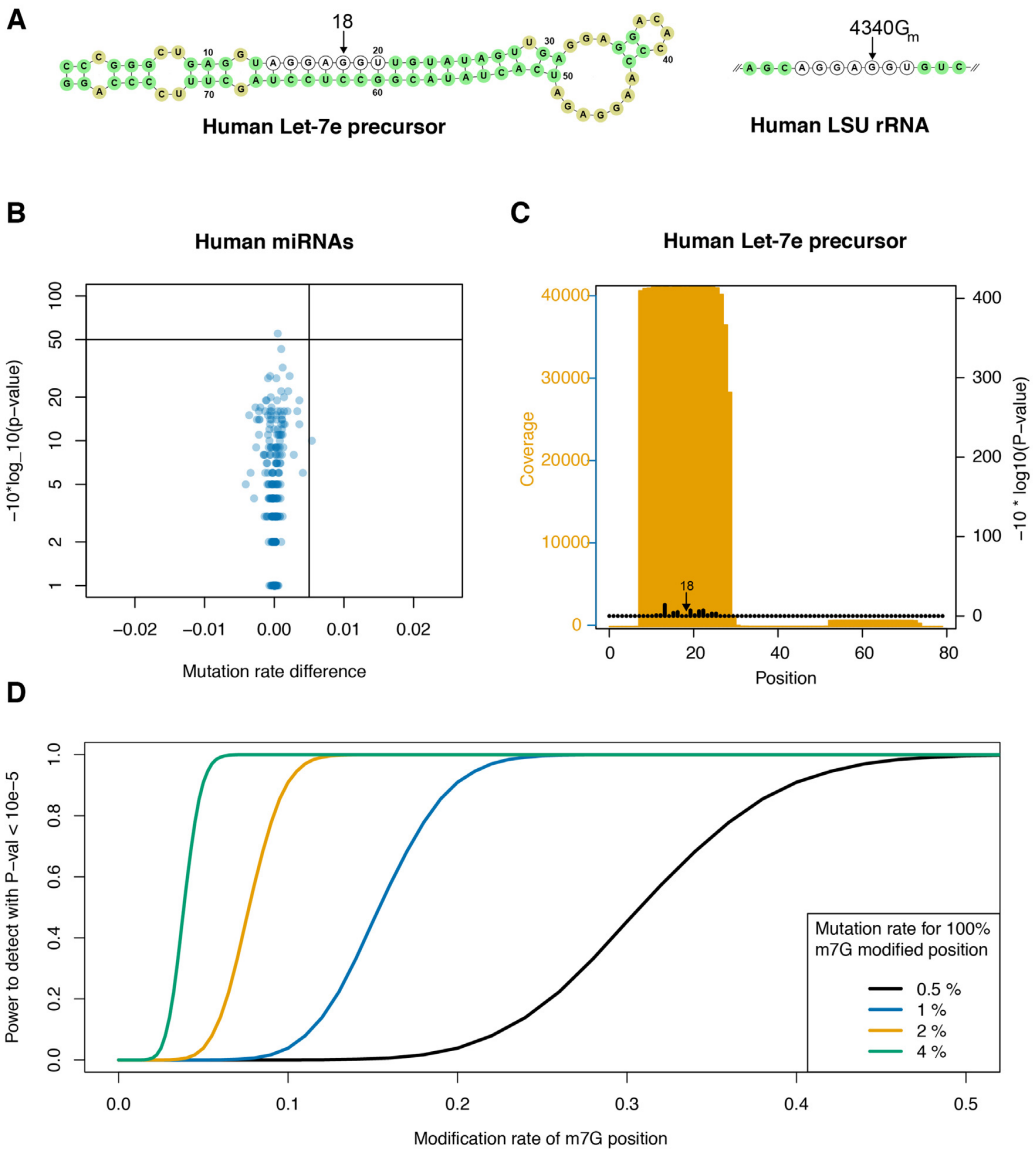
(**A**) Sequences of the Human Let-7e precursor and human LSU rRNA 4333-4345. Identical RNase A fragments are indicated as well as the Let-7e position reported by Pandorfini *et al.* to be m7G modified and the corresponding 2’ OMe G4340 position known to be modified in LSU rRNA and guided by snoRNA U60 (Krogh *et al.*). (**B**) Mutation rate difference versus –10*log(*P*-val) plot for 1239 Gs in 175 different human miRNAs. (**C**) Coverage and *P*-values obtained for the Human Let-7e precursor sequence. Position 18 is indicated. (**D**) Analysis of the power to detect m7G modification at position 18 in the human Let-7e. The curves show the power for 4 different detection levels of m7G modifications with the sequencing depth observed for Let-7e.

A subset of yeast tRNAs undergoes splicing and it is not known whether these tRNAs are m^7^G modified before or after splicing takes place. We therefore compared the m^7^G-MaP-Seq signal from the reads mapping to the unspliced and the mature tRNA, respectively. As previously demonstrated for tRNA m^1^A modifications, we find that the unspliced yeast tRNA molecules are m^7^G modified on position 46 prior to splicing, suggestion that m^7^G modification take place early in tRNA maturation (Supplementary Figure S6). Next, we applied m^7^G-MaP-Seq to small RNA isolated from human HeLa cells and arabidopsis (Figure 4). Human and arabidopsis tRNAs have more RT-terminating modifications such as m^1^A, *N*^3^-methylcytidine (m^3^C) and *N*^1^-methylguanosine (m^1^G) and *N*^2^,*N*^2^-dimethylguanosine (m^2^_2_G), resulting in relatively fewer tRNAs having sufficient sequencing depth for analysis. Nevertheless, among the tRNAs expressed in HeLa cells, m^7^G-MaP-seq identifies eight different tRNA isodecoder families modified at position 46 (Figure 3D), for all of which the homologous mouse tRNA were previously found to be m7G modified by the TRAC-seq method (12). For tRNA-Val-CAC, we find that isodecoders 1, 2 and 3 are all m^7^G modified on position 46, whereas the isodecoder 11 did not show any evidence of modification in m^7^G-MaP-seq.

There is no available annotation of m^7^G tRNA modifications in arabidopsis and we therefore applied m^7^G-MaP-seq to small RNA purified from arabidopsis 14-day old seedlings (Figure 3E). Based on the analysis, we predict that Arg−ACG, Asn−GTT, Asp−GTC Met−CAT, Pro−TGG, Thr−TGT, Trp−CCA−3, Thr−GGT and Thr−TGT are m^7^G modified, whereas Arg-TCT, Glu-TTC and Leu-TAG are not modified.

### No evidence for m^7^G modifications in human miRNAs, including human Let-7e

Kouzarides and colleagues recently reported m^7^G modifications within a subset of human miRNAs, including Let-7e, for which the m^7^G modification was validated and localized to position 18 using mass spectroscopy of a RNaseA fragment of the miRNA (Figure 4A) (23). While the application of m^7^G-MaP-seq to human small RNAs mapped known tRNA m^7^G modifications with high confidence (see Figure 3), we find no evidence of m^7^G modifications in any human miRNA (Figure 4B). For Let-7e, we have very high sequencing depth (Figure 4B) but we do not observe any mutations in the treated samples for this position. In this specific experiment, tRNAs m^7^G positions are detected with mutational rates in the range of 0.5% to 6% (Figure 3D). If we conservatively assume that the m^7^G modification on Let-7e would also be detected with a 1% mutation rate if 100% modified, then we estimate that we would have 100% power to detect the Let-7e modification with a *P*-value better than 10^−5^ if 10% of Let-7e molecules were m^7^G modified (Figure 4C). The fact that we do not observe any mutations for Let-7e position 18 demonstrates that for the HeLa cells used in our study, this position is not m^7^G modified at a biologically relevant level.

### No evidence for m^7^G modifications in yeast and E. coli mRNAs

**Figure 5.**
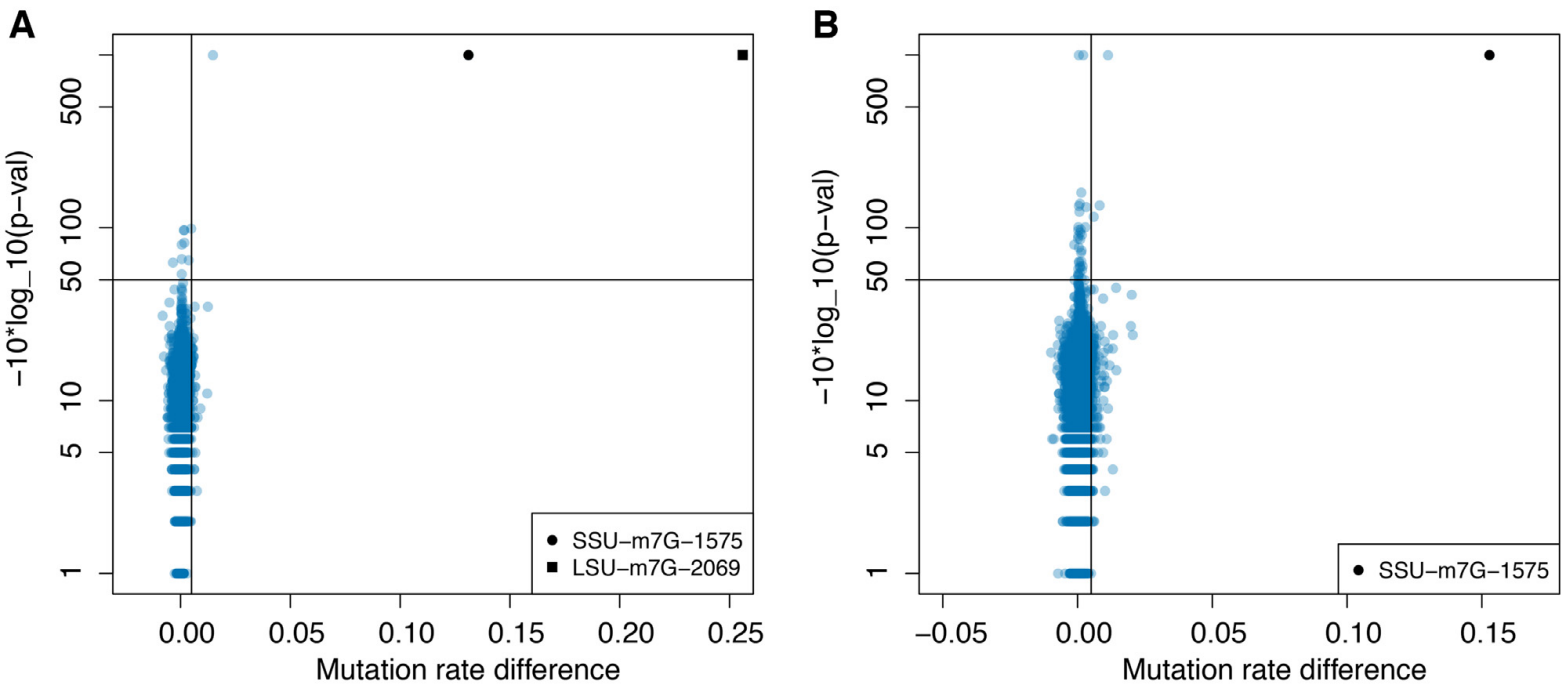
(**A**) Mutation rate difference versus –10*log_10(*P*-val) plot for *E. coli* mRNA G positions (*n* = 193 590) and *E. coli* rRNA G positions. The two known *E. coli* ribosomal m7G modifications are indicated. (**B**) Mutation rate difference versus –10*log_10(*P*-val) plot for yeast mRNA positions (*n* = 377 837). The known yeast ribosomal m7G modification at 1575 is indicated.

In human mRNAs, 803 m^7^G modified positions were recently reported, suggesting that mRNA in other species may also be m^7^G modified. We applied m^7^G-MaP-seq to mRNA from *E. coli* and yeast in biological triplicates. In our analysis, 193590 *E. coli* mRNA (Figure 5A) and 377837 yeast mRNA G positions (Figure 5B) had a sequencing depth of more than 1500 in the three control and three treated replicates combined, an average mutation frequency <1% in the control replicates, and no significant difference in mutation rates within replicates (see Materials and Methods for details). This corresponds to approximately 20% and 16% of the G content of the entire transcriptome of *E. coli* and yeast, respectively. However, in neither of the two analyses, we find any evidence of internal mRNA positions being m^7^G modified. In contrast, we find the known ribosomal m^7^G modifications at *E. coli* SSU 527 and LSU 2069 (Figure 5A) and yeast SSU 1575 (Figure 5B) are detected with high mutation rate differences and high confidence.

## DISCUSSION

In this paper, we present m^7^G Mutational Profiling sequencing (m^7^G-MaP-seq), which allows sensitive and high throughput detection of internal m^7^G modifications in any type of RNA. Mutational profiling has previously been applied to obtain nucleotide resolution mapping of RNA-protein interactions, m^1^A modifications and for Selective 2-hydroxyl acylation analysed by primer extension (SHAPE) based probing of RNA structure, and here we demonstrate that this strategy also can be successfully applied to detect internal m^7^G modifications. Our method is based on the treatment of RNA with NaBH_4_, leading to the formation of abasic sites at m^7^G positions. Abasic sites in RNA are relatively stable, and during the subsequent reverse transcription m^7^G positions are permanently recorded as mutations in the cDNA sequence. m^7^G-MaP-seq can be implemented in any sequencing protocol, including regular RNA-seq experiments, and small RNA-seq by addition of NaBH_4_ treated samples in the experimental setup and application of our pipeline for data analysis. Compared to methods based on the mapping of reverse transcription stops, mutational profiling makes better use of the sequencing reads and has improved sensitivity because bias from RNA fragmentation, adapter ligation and secondary structures is reduced. In global RNA modification mapping experiments, it becomes very important to establish the background detection rates and account for these when modifications are mapped (33). We developed a pipeline that incorporates re-estimated base qualities, which are used to provide estimated mutation frequencies using an EM-algorithm. In addition, our tool applies log likelihood ratio tests to obtain *P*-values for differences in mutation rates both within replicates and between the control and NaBH_4_ treated samples, making m^7^G-MaP-seq highly suitable for global analyses.

Apart from m^7^G, we also find that NaBH_4_ treatment induces increased mutation rates of N4-acetylcytidine (ac^4^C), dihydrouridine (D) and m^1^A. This is in agreement with a recent publication using NaBH_4_ treatment for detection of ac^4^C (34) and the known susceptibility of D for cleavage of the dihydrouridine ring upon reduction by NaBH_4_ (32). In contrast, for m^1^A positions NaBH_4_ treatment was not expected to lead to increased mutation rates. Rather, the alkaline conditions during reduction could potentially lead to Dimroth rearrangement to m^6^A and thereby decrease mutation rates, as previously used to improve m^1^A signal in global sequencing-based mapping experiments (15). Typically, the Dimroth rearrangement is performed at elevated temperatures (35), whereas we here perform NaBH_4_ treatment on ice, which likely explains that we do not observe widespread rearrangement to m^6^A and decreased mutation rates. NaBH_4_ treatment of m^1^A results in the formation of 1-methyl-6-hydroadenosine, which is fairly stable, but can be re-oxidized to m^6^A (35). The reduction to 1-methyl-6-hydroadenosine results in the loss of planar conformation and aromatic character of the six-membered ring. Our results suggest that 1-methyl-6-hydroadenosine is relatively stable under our conditions and that A is relatively efficiently incorporated into cDNA during reverse transcription at 1-methyl-6-hydroadenosine positions (Supplementary Figure S5B).

We find that different m^7^G positions have variation in their m^7^G-MaP-seq mutational rates, which may be the result of differences in the level of m^7^G modification, but probably also reflects that the local sequence influences the efficiency of abasic sites formation during reduction and of reverse transcriptase abasic site read-through. In this study, we successfully use MMLV based reverse transcriptases Primescript (total RNA and mRNA libraries) and Protoscript II (small RNA libraries) for m^7^G-MaP-seq, however, the use of thermostable group II intron reverse transcriptase (TGIRT) could potentially allow higher read-through rates and thereby increase the signal. Another possibility could be inclusion of Mn^2+^ in the reverse transcription buffer, which in SHAPE-MaP-seq has been shown to increase RT read-through rates of 2′ acetylated RNA positions (36). While the study was under review, He *et al.* published the m^7^G-seq method, which also use mutation mapping for detection of m^7^G modifications. Compared to our method m^7^G-seq includes an additional step to biotinylate the abasic sites, which allows subsequent enrichment of the RNA fragments. For this reason, m^7^G-seq will probably be better to identify low frequency m^7^G modification than m^7^G-MaP-seq. In future applications of m^7^G-MaP-seq, we plan to combine our protocol with m^7^G specific antibody immunoprecipitation of RNA fragment (12,13,23) before the NaBH_4_ treatment, which potentially would facilitate the detection of positions with low frequency m^7^G modifications.

We demonstrate that m^7^G-MaP-seq detects known and novel m^7^G modifications in rRNA and tRNA. In human SSU rRNA, we detect the modification of position 1639, which has previously been shown to be m^7^G modified by WBSCR22-TRMT112 (4,5). This modification is conserved to yeast SSU position G1575 and here we show that the arabidopsis SSU rRNA is also modified at the homologous position G1581 (Figure 2C), suggesting that SSU m^7^G methylation is conserved between several eukaryotes and that arabidopsis Bud23 homolog RID2 is a functional m^7^G methyltransferase (37). In contrast, we did not observe any signal at position 1605, 2522 and 4550 in human LSU rRNA, which were recently reported to be m^7^G modified based on a high resolution cryoEM structure of the human 80S ribosome (38). As in our study, the cryoEM study is based on rRNA purified from HeLa cells, suggesting that the inconsistent results are not cell type related. Our data, together with mass spectroscopy based analysis of human LSU rRNA from the Isobe group (39), therefore strongly suggests that m^7^G modifications are not reliably detected from current cryoEM data, which is furthermore supported by the fact that the cryoEM study did not detect the well-known m^7^G modification on SSU position 1639 (38).

We also applied m^7^G-MaP-seq to small RNA purified from yeast, arabidopsis and human cells. In these experiments, we observe m^7^G modifications on tRNAs only, for which a subset is known to be m^7^G modified at position 46 in the variable loop, whereas no other sRNA was found to be modified (Supplementary Figure S7). tRNA modifications have been most extensively annotated in yeast and with m^7^G-MaP-seq we validate nine out of twelve tRNA previously known to have m^7^G modifications in yeast. In addition, we demonstrate m^7^G modifications on tRNA-Pro-AGG and tRNA-Thr-TGT, which were not previously known to be m^7^G modified. Our results are in agreement with existing mapping of yeast m^7^G modifications, including tRNAs previously found not to be m^7^G modiifed (Supplementary Table S3) (11). By specifically analyzing the reads containing tRNA introns, we find that for tRNA m^7^G modifications occur early, before the tRNA intron has been spliced out (Supplementary Figure S6), similar to what was previously shown for m^1^A modifications (20). Among human tRNAs, we identify m^7^G modifications on 8 different isodecoder families (Figure 4). Gregory and colleagues previously published the TRAC-seq method and applied it to small RNA from mouse embryonic stem cells (12). Their study implemented AlkB treatment to increase sequencing depth of tRNA carrying modifications that terminate reverse transcription and convincingly identified the entire subset of mouse tRNA that are m^7^G modified. Likewise, He and colleagues applied their m^7^G-seq method to detect m^7^G modifications human tRNA. Our results on human tRNA agree with the TRAC-seq and m^7^G-seq data, but we have sufficient sequencing depth on much fewer tRNAs. Interestingly, our m^7^G-MaP-seq data shows that human tRNA-Val-CAC-11 is not m^7^G modified, whereas tRNA-Val-CAC-1, 2, 3 and 4 are all predicted to be m^7^G modified, although all these Val-CAC isodecoders have exactly the same variable loop sequence. However, Val-CAC-11 has a destabilized T-arm with one mismatch and three G-U wobble basepairs, which may explain the lack of modification as the T-arm was previously shown to be required for m^7^G modification of yeast tRNA-Phe by purified Trm8–Trm82 heterodimer (40). These results show the potential of m^7^G-MaP-seq to distinguish tRNA isodecoders and determine their m^7^G modification status. For arabidopsis tRNAs, we provide a preliminary annotation of m^7^G modifications, where 9 tRNAs were predicted to be modified. Future implementation of AlkB treatment and/or TGIRT in the m^7^G-MaP-seq protocol should facilitate a complete annotation of m^7^G tRNA modifications in arabidopsis and for most other species for which no m^7^G modification annotation exists.

In a recent publication by Pandolfini *et al.*, it was reported that a subset of human miRNAs are m^7^G modified, including Let-7e, for which the m^7^G modification was validated and localized to position 18 using mass spectroscopy of a RNase A fragment of the gel purified miRNA fraction (Figure 5A) (23). In HeLa cells, we demonstrate that Let-7e position is not m^7^G modified and we note that the sequence of the Let-7e RNase A fragment analysed by Pandolfini *et al.* is identical to the human LSU rRNA 4336–4342 RNase A fragment (Figure 5A). The LSU rRNA 4336–4342 fragment has previously been analysed with mass spectroscopy and was shown to be 2′ OMe modified on position 4340 (39), which is in agreement with results from sequencing-based 2′ OMe mapping experiments on human ribosomal RNA (41). LSU rRNA position 4340 and Let-7e position 18 are both is position 5 in the RNase A fragments (Figure 5A), meaning that the mass spectra obtained by Pandolfini and colleagues for Let-7e m^7^G modification potentially could be derived from rRNA contamination of the miRNA fraction. Alternatively, the A549 cells used in their study may have more abundant miRNA m^7^G modifications than the HeLa cells used in our study.

Zhang *et al.* used mass spectroscopy on purified and decapped mRNA fractions from different human mouse cell types and found that the mRNAs contained from 0.02 to 0.05% m^7^G when compared to the amount of G (13). They also used m^7^G-seq to detect 803 human mRNA internal m^7^G modifications that were detected in both HeLa and HepG2 cells. We applied m^7^G-MaP-seq to mRNAs purified from *E. coli* and *S. cerevisiae* cells and did not identify any m^7^G modified internal mRNA positions, whereas we in the same experiment detect the known ribosomal m^7^G modifications with high significance (Figure 5). For both species, our analysis has high sequencing depth on a substantial fraction of Gs in the transcriptome, 20 and 16 percent, for *E. coli* and *S. cerevisiae*, respectively. We estimate that the cut-off sequencing depth of 750 reads per group (1500 in total) gives us around 90% power to detect a m^7^G modification present at a frequency of 0.2, whereas a sequencing depth of 3000 reads per group gives 90% power to detect a modification present at a frequency of 0.05 (Supplementary Figure S8). We therefore demonstrate that medium/high frequency internal m^7^G modifications are not widespread in *E. coli* and *S. cerevisiae* mRNA. Future application of m^7^G-seq or m^7^G-MaP-seq with higher sequencing depth and potentially combined with antibody mediated m^7^G pull-down will allow detection of potential low frequency m^7^G modifications in highly expressed mRNA and high frequency m^7^G modifications in lowly expressed mRNA.

In conclusion, we have developed the m^7^G-MaP-seq method, which allows high throughput detection of m^7^G RNA modifications. The method can be implemented with existing RNA-sequencing protocols and we provide an efficient computational pipeline for data analysis. In this study, we identify known and novel m^7^G modifications in rRNA and tRNA and find that the human Let-7e is not m^7^G modified in HeLa cells. We expect that the m^7^G-MaP-seq method will facilitate future studies on internal m^7^G RNA modifications.

## Supplementary Material

gkz736_Supplemental_File

## Notes

Present address: Søren Iversen, Department of Bacteria, Parasites & Fungi, Statens Serum Institut, 2300 Copenhagen S, Denmark.
