# Supplementary material for: Detection of internal N7-methylguanosine (m7G) RNA modifications by mutational profiling sequencing
Source: Nucleic Acids Res. 2019 Aug 31;47(20):e126. doi: 10.1093/nar/gkz736 (PMC6847341; doi:10.1093/nar/gkz736)
Supplement: gkz736_Supplemental_File [file gkz736_supplemental_file.pdf]

Supplementary information for

Detection of internal N7-methylguanosine (m^7G) RNA modifications by mutational profiling sequencing

Christel Enroth[1,4], Line Dahl Poulsen[1,4], Søren Iversen[1], Finn Kirpekar[3], Anders Albrechtsen[1], Jeppe Vinther[1]*,

[1] Department of Biology, University of Copenhagen, Ole Maaløes Vej 5, DK-2200 Copenhagen N, Denmark
[2] Department of Biochemistry and Molecular Biology, University of Southern Denmark, Campusvej 55, DK-5230 Odense M, Denmark

* To whom correspondence should be addressed. Tel: +45 35 32 12; Email: jvinther@bio.ku.dk

[3] The authors wish it to be known that, in their opinion, the first 2 authors should be regarded as joint First Authors.

Present Address: Søren Iversen, Department of Bacteria, Parasites & Fungi, Statens Serum Institut, 2300 Copenhagen S, Denmark.

CONTENT

Supplementary methods: Calculation of mutation frequencies and statistical analysis

Supplementary figure 1: m7G-MaP-seq raw sequencing data

Supplementary figure 2: m^7G-MaP-seq validation

Supplementary figure 3: Supplementary figure 2: Stop-rate analysis

Supplementary figure 4: Tandem mass spectrometry of Arabidopsis SSU position 1578-1584

Supplementary figure 5: m1A sensitivity to $NaBH_4$ treatment

Supplementary figure 6: m^7G modifications in precursor and mature tRNA

Supplementary figure 7: Analysis of sRNA and miRNA m^7G modification

Supplementary figure 8: Power analysis of mRNA m^7G detection by M^7G-MaP-seq

Supplementary table 1: Oligonucleotides used in this study

Supplementary table 2: Explanation of output from getFreq function

Supplementary table 3: m^7G tRNA modifications in yeast

Supplementary references

Supplementary methods: Calculation of mutation frequencies and statistical analysis

We use the mpileup function from the samtools package to compile sequencing results for each base position present in the sequence file that was used for mapping. For each position, this function summarises the sequencing data into the observed bases with their corresponding quality score for each sample (1,2). We developed the getFreq tool to estimate the mutation frequencies for each position by taking the observed sequenced bases and possibility of sequencing/alignment error into account.

Formal description of the estimation of mutation frequencies

For each position, we first removed bases/indels that are observed at very low frequency (default=0.1%) or bases/indels that are only observed a low number of times (default=1). For the remaining bases/indels, we estimated the allele frequency based on a likelihood function that includes the possibility of sequencing error modelled based on the base quality score.

$$\hat{f} = argmax_f \; P(X|f),$$

where X is the sequencing data for the position and f in the frequency of the possible alleles including indels e.g. f=(f_A,f_G,f_{AGG}) if both A, G and indel AGG is observed at this loci. The likelihood assumes independence per read such that for N reads

$$P(X|f) = \prod_{i=1}^{N} P(X_i|f).$$

The probability of observed the sequencing data for a single read depends on the true allele that was sequenced and we assumed that the true allele, A, is one of the T observed ones such that

$$P(X_i|f) = \sum_{j=1}^{T} P\left(X_i \middle| A = a_j\right) p(A = a_j|f)$$

Where we sum over all of the possible true alleles a_j. The probability of $p(A = a_j|f)$ is simply the frequency of the a_j allele. To obtain the probability of the sequencing data of the ith read we convert the quality score in to a probability of error, e, based on the Phred scaling. We assumed that if we observe allele of type b that

$$P\left(X_i = b \middle| A = a_j\right) = \begin{cases} 1 - e, & if\ b = a_j \\ e/3, & otherwise \end{cases}$$

Where the division of 3 is motivated by the fact that sequencing error of a base can results in three other bases with equal probability. Since we also allow for indels this should be seen as an approximation.

Formal description of the calculation of p-values for positions having mutations:

To test if a position is polymorphic (has a significant amount of mutations), we calculated the likelihood based on the estimates allele frequency $\hat{f}$and under the null where all alleles are the same, f_0, using a likelihood ratio statistics

$$\boldsymbol{Y} = \boldsymbol{2log}\left(\frac{P(X|\hat{f})}{P(X|f_0)}\right).$$

If there are T types of possible alleles then $Y \sim \chi^2_{T-1}$, where T-1 is the number of degrees of freedom. When multiple samples are analysed, we can test for differences between samples or groups of samples by estimating the frequencies jointly and separately in the groups. The test statistics then become

$$\boldsymbol{Y} = \boldsymbol{2log}\left(\frac{\prod_{k=1}^{k=K} P(X^k|\widehat{f^k})}{P(X|\hat{f})}\right)$$

Where X^k is the sequencing data in group k of K groups, f^k is the allele frequency in group k. Here we will have (K-1)*T degrees of freedom.

Outputs from the getFreq function:

For each position, the getFreq function reports the estimated mutation frequencies for the control (AltFreqCC0) and treated (AltFreqCC1) samples as well as the mutation rate difference (relFreq). Moreover, the getFreq function reports p-values that have been log10 transformed and multiplied by -10. CCPval is the significance for there being a difference between the mutation rates observed in the treated and the control samples. The indPval is a p-values for each of the analysed samples testing whether the sample has a mutation frequency different from zero. For m7G positions, the individual p-values should be significant for treated samples, but not controls. CC0Pval and CC1Pval are the p-values for testing whether the observed mutation rates are different within the control and treated group, respectively. Preferably, the mutation rates obtained within the group should not be significantly different. Finally, the SNPPval in the p-value for a given position having a non-zero mutations frequency taking all samples into account. For a full description of the getFreq output see Supplementary table 2.

Supplementary figure 1: m7G-MaP-seq raw sequencing data

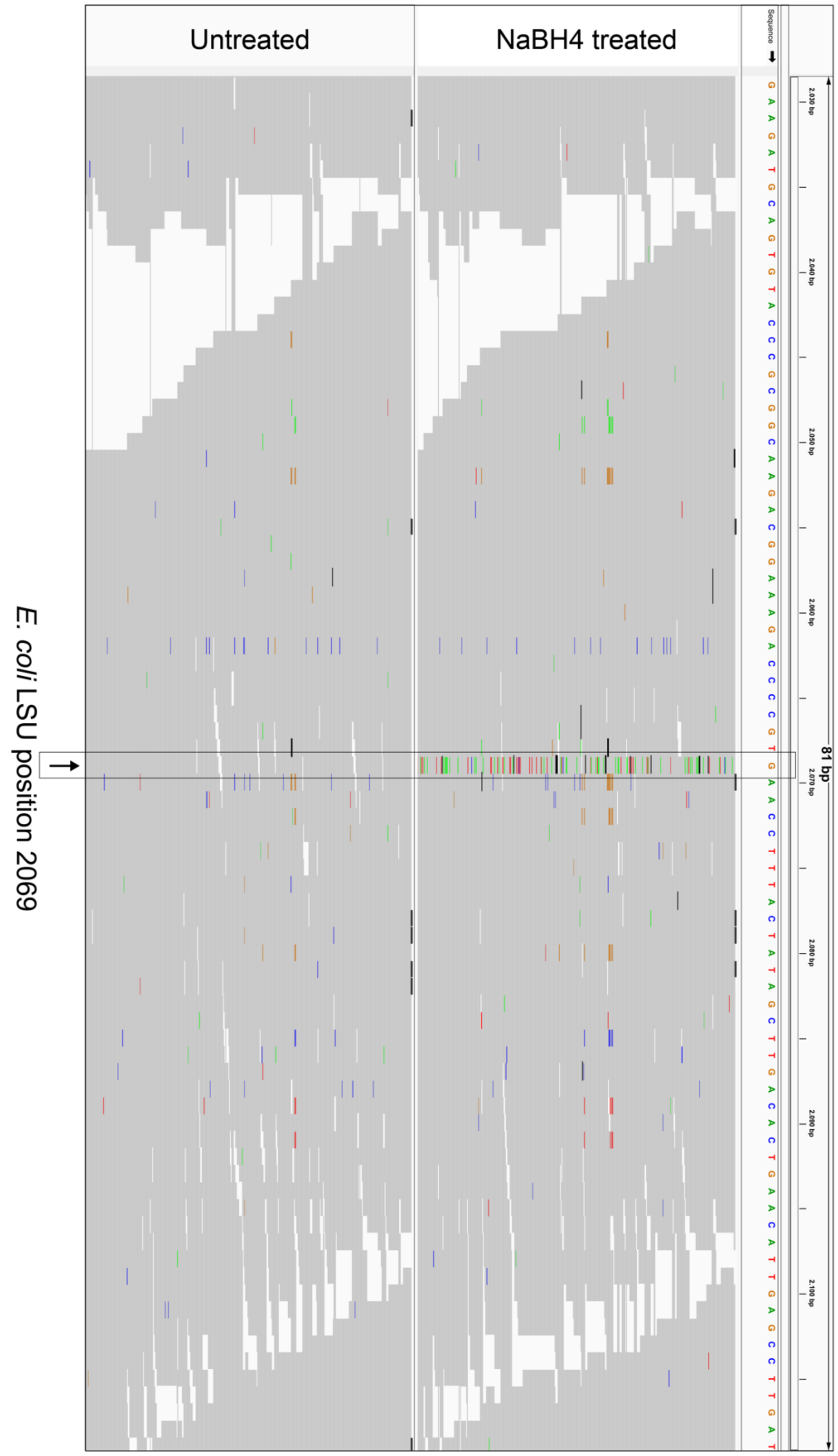

Supplementary figure 1: m7G-MaP-seq raw sequencing data
IGV browser view of 200 random m7G-MaP-seq reads from a NaBH4 treated and a control sample. The reads cover *E. coli* LSU position 2069, which is known to be m7G modified.

Supplementary figure 2: m7G-MaP-seq validation

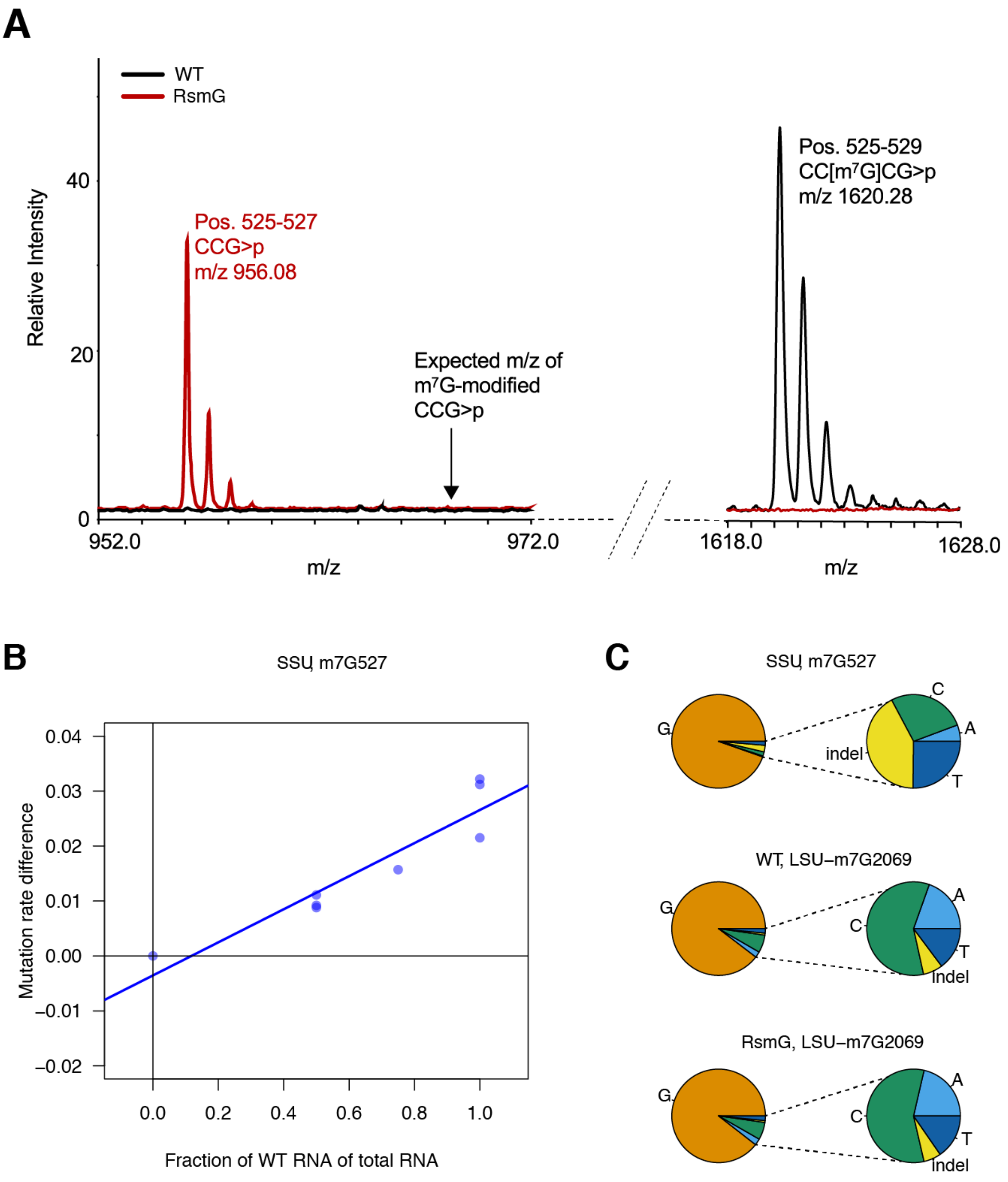

Supplementary figure 2: m7G-MaP-seq validation

A) Overlaid MALDI mass spectra of *E. coli* 16S rRNA (position 500-549) digested with RNase T1; only m/z regions relevant to the m^7G527 modification are shown. Black trace: WT (strain BW25113); Red trace: In-frame RsmG deletion strain. m7G modification completely precludes RNase T1 cleavage resulting in the CC[m^7G]CG>p product in the wild type with a negligible signal for CCG>p, suggesting nearly complete methylation of G527. The RsmG deletion strain reveals the expected signal patter with G527 only being present in a CCG>p context. The intensity scale applies to all m/z traces. B) m7G-MaP-seq was applied to a mixtures of RNA isolated from the WT and the RsmG deletion strain and the mutational rate difference was calculated. C) Mutational signature of SSU rRNA m7G527 in the WT strain and LSU rRNA m7G2069 in the WT and RsmG strains.

Supplementary figure 3: Stop-rate analysis

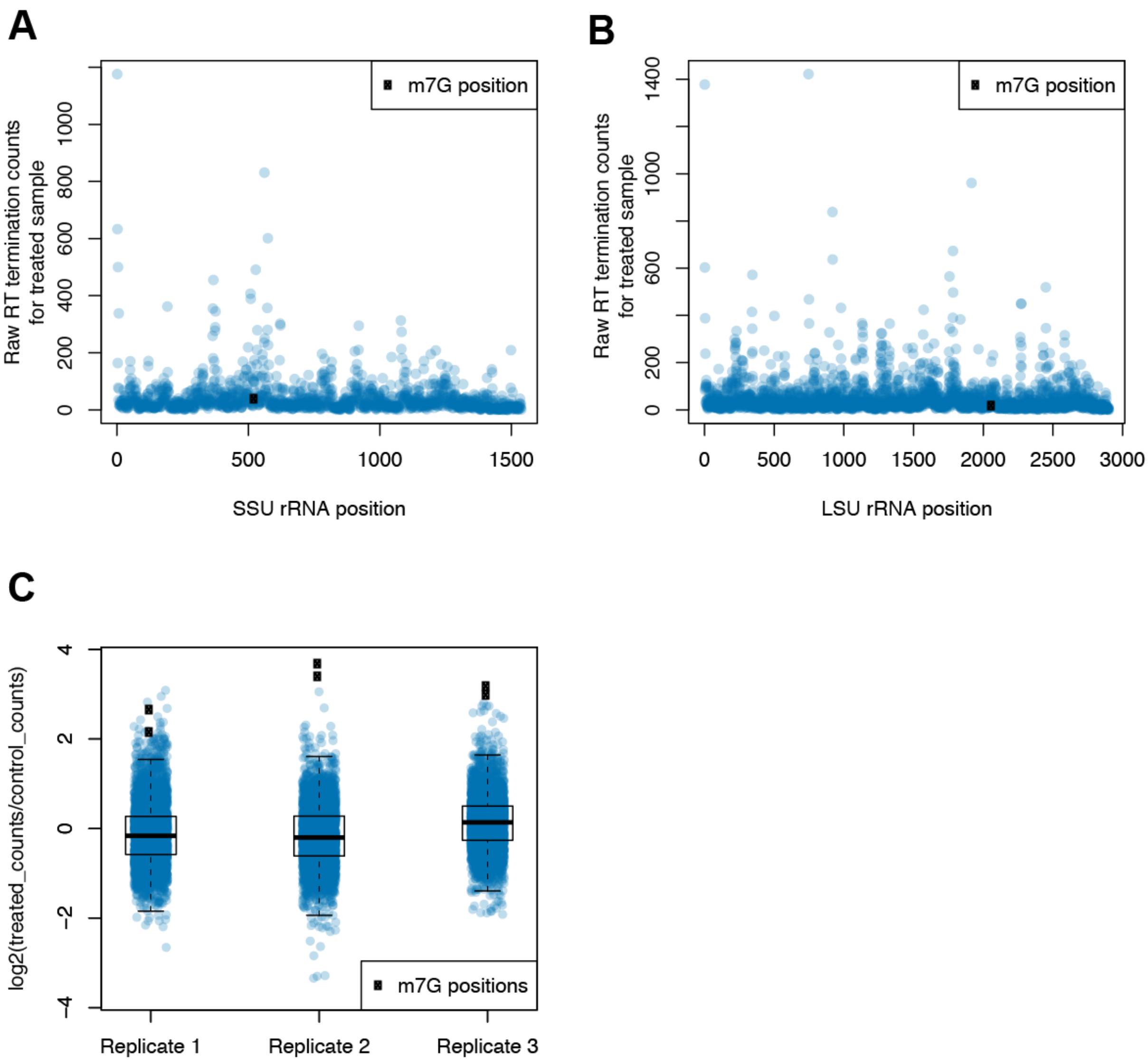

Supplementary figure 3: Stop rate analysis

Plot of the raw reveres transcription termination counts (collapse on barcodes to remove potential PCR duplicates) for $NaBH_4$ treated sample across A) *E. coli* SSU rRNA and B) LSU rRNA as determined by the RNAprobR R package. C) Log2 ratio of counts from $NaBH_4$ treated samples divided by counts from control samples for the same three replicates shown in Figure 1D). The analysis in D was performed as previously described using barcode counts (3).

Supplementary figure 4: Tandem mass spectrometry of Arabidopsis SSU position 1578-1584

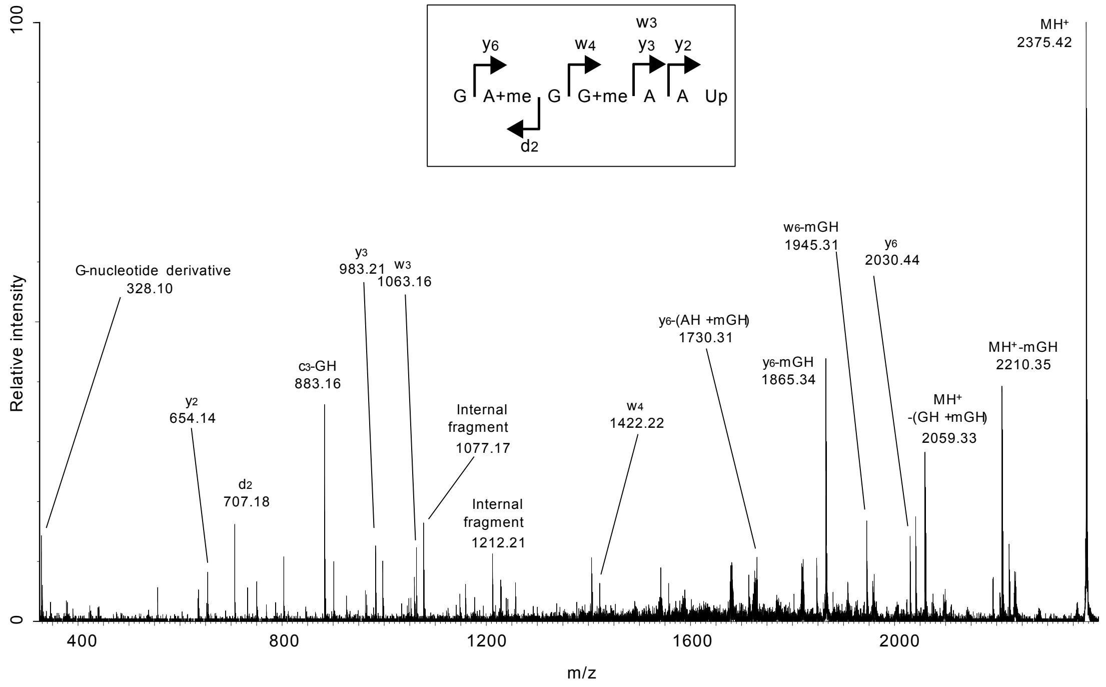

Supplementary figure 4: MALDI Tandem mass spectrum of expected di-methylated RNase A product of *A. thaliana* SSU rRNA (position 1578-1584). Sequence (backbone cleavage) ions and other major signals are assigned. The insert places the observed sequence ions onto the expected RNase A product. MH^+: Parent ion selected for tandem MS. AH: adenine. GH: guanine. mGH: methylated guanine. a, b, c, d and w, x, y, z: 5' and 3' backbone fragment ions, respectively. Digit in subscript indicates number of nucleotides in fragment. According to nomenclature in (4).

Supplementary figure 5: m1A sensitivity to $NaBH_4$ treatment

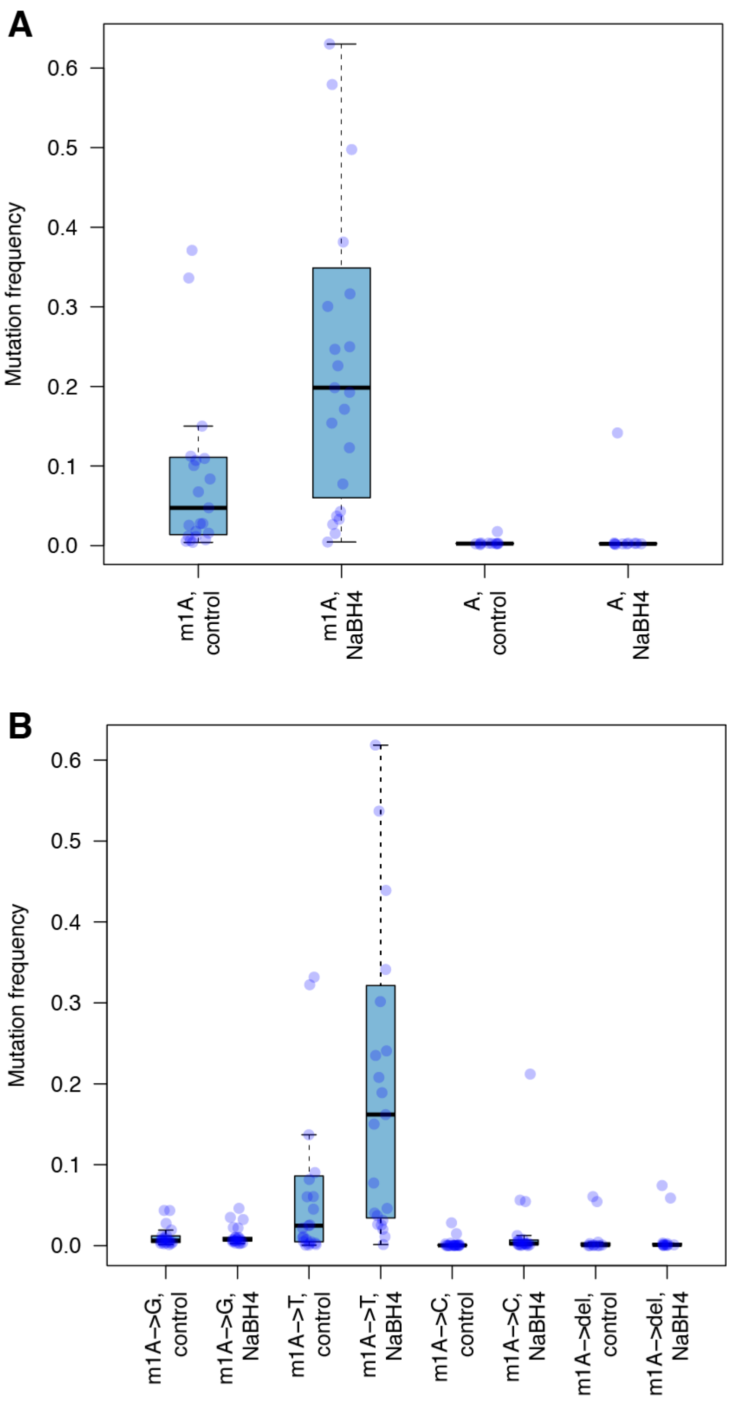

Supplementary figure 5: m1A sensitivity to $NaBH_4$ treatment

A) Mutational frequencies for yeast tRNA A positions and annotated m1A positions (5) in the control and $NaBH_4$ treated samples. B) Specific mutation frequencies observed for yeast tRNA annotated m1A positions (Modomics database). The figures show all A positions having sequencing depths of more than 1500 and no significant difference in mutation rates (p-value < 10^{-5}) within the control or NaBH4 treated replicates.

Supplementary figure 6: m^{7}G modifications in precursor and mature tRNA

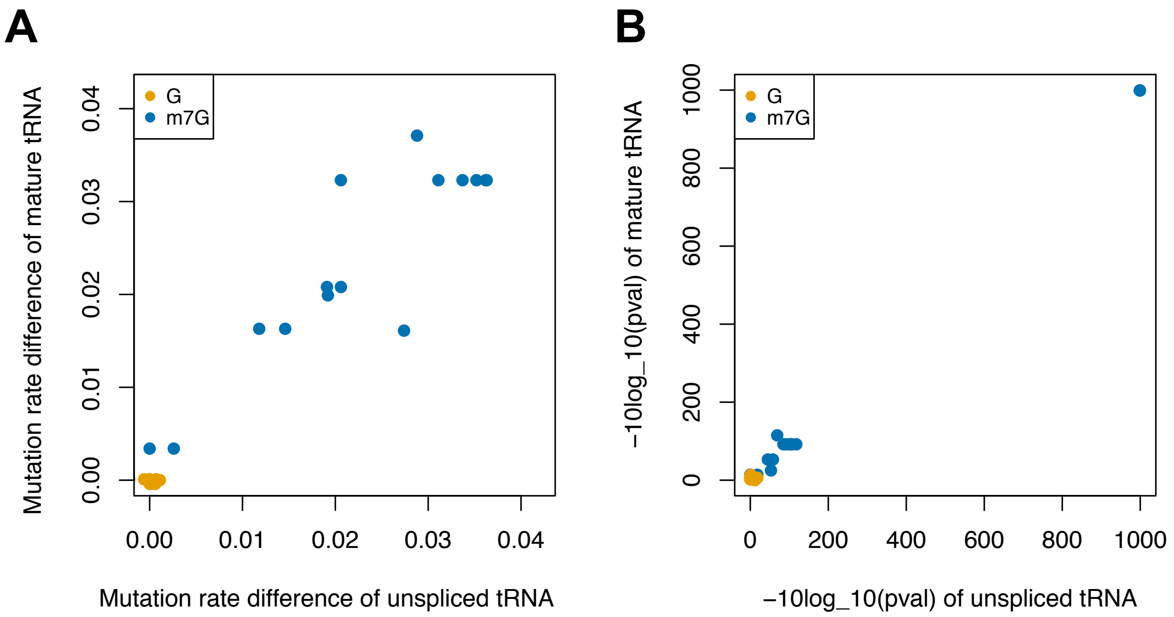

Supplementary figure 6: m^{7}G modifications in precursor and mature tRNA. A) Mutation rate difference between the control and the NaBH4 treated samples observed for mature tRNAs known to have either m^{7}G or G in position 46 in the variable loop and the corresponding unspliced precursor tRNA. For some mature tRNAs, several different genes exist. B) P-values for mutation rate difference between the control and the $NaBH_4$ treated samples observed for mature tRNAs known to have either m^{7}G or in position 46 in the variable loop and the corresponding unspliced precursor tRNA. For some mature tRNAs, several different genes exist.

Supplementary figure 7: Analysis of sRNA and miRNA m7G modification

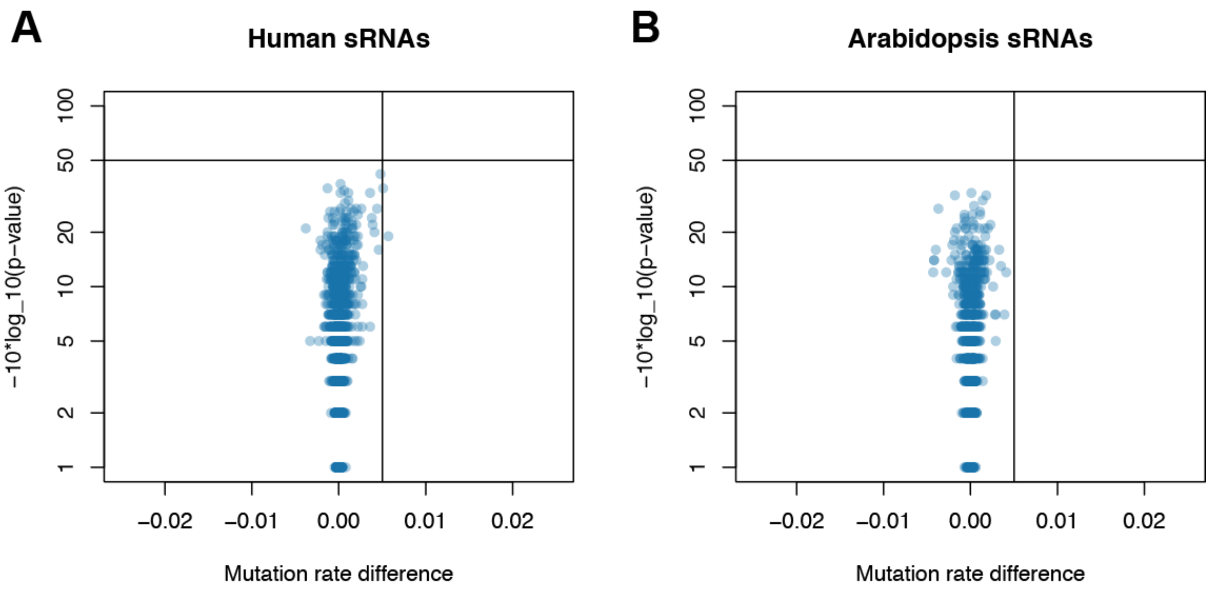

Supplementary figure 7: Analysis of sRNA m^7G modifications. A) Small RNA reads were mapped to human snoRNAs and sRNAs. 4184 Gs in 245 different sRNAs passed the cut-offs described in the methods section. B) Small RNA reads mapped to arabidopsis snoRNAs and sRNAs. 3315 Gs in 217 different sRNAs passed the cut-offs described in the methods section.

Supplementary figure 8: Power analysis of mRNA m^7G detection

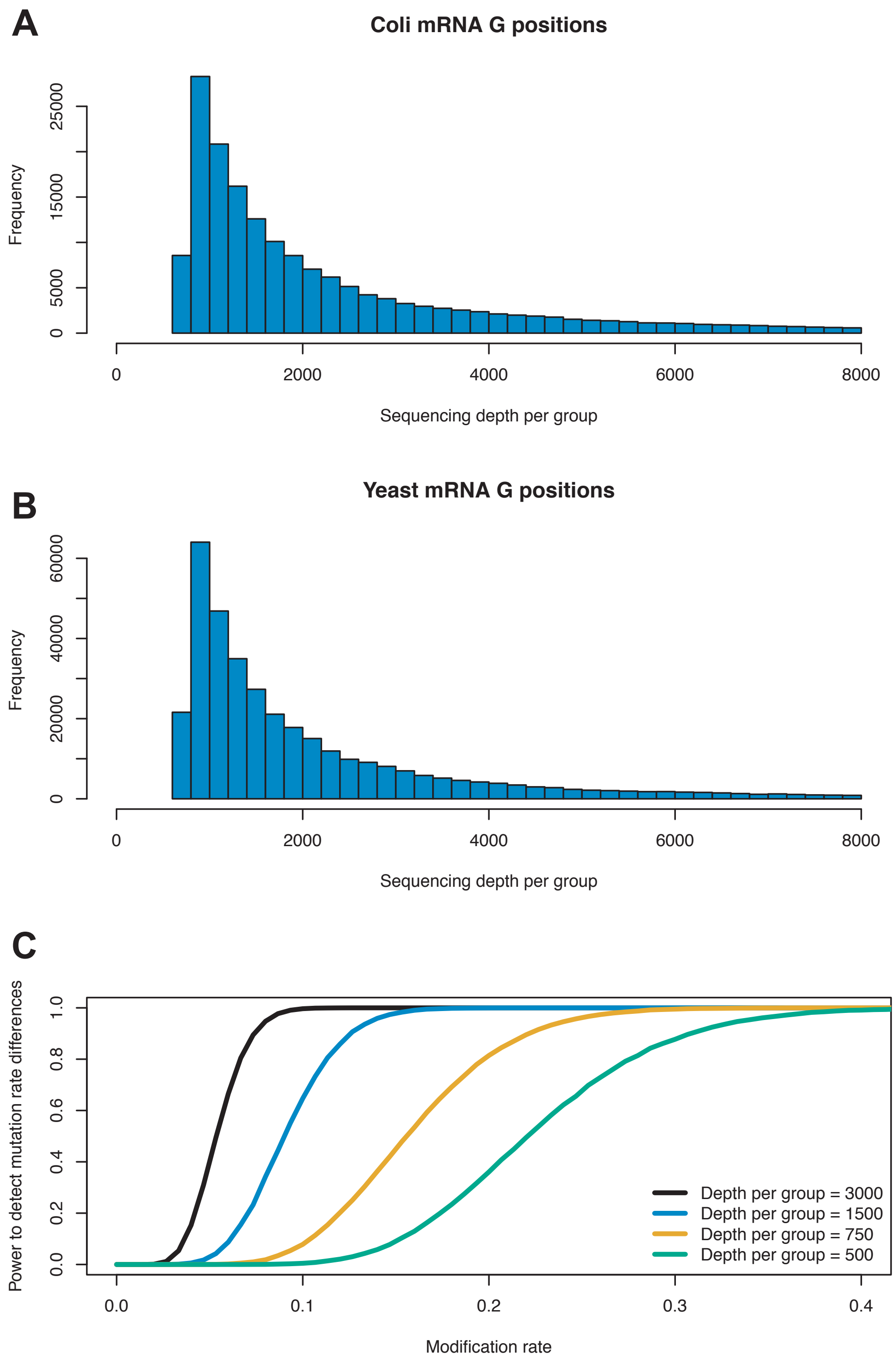

Supplementary figure 8: Power analysis of mRNA m^7G detection. A) Sequencing depth of Gs analysed in E. coli mRNA experiment. B) Sequencing depth of Gs analysed in yeast mRNA experiment. C) Simulated power to detect an m^7G modification with a p-value of 10^{-5} for 4 different sequencing depths. The simulations assume that a 100% modified position has a 15% mutation rate (as observed for ribosomal m7G RNA modifications in this experiment), sequencing depth is equal in the two groups, errors gives rise to the same observed base and a base error rate of 0.1 %.

Supplementary table 1: Oligonucleotides used in this study

Oligo name	Oligo sequence (5'- 3')
**Ara_LSU_1581**	GATGACTCGCGCTTACTAGGAATTCCTCGTTGAAGACCAACAATTGCAATGA
**RT_random_primer**	AGACGTGTGCTCTTCCGATCTNNNNNNNNNNNNNNN
**Ligation_adapter**	PHO-NNNNNNNAGATCGGAAGAGCGTCGTGTAGGGAAAGAGTGT-3NHC3
**PCR_forward**	AATGATACGGCGACCACCGAGATCTACACTCTTTCCCTACACGACGCT
**PCR_reverse_index1**	CAAGCAGAAGACGGCATACGAGATCGTGATGTGACTGGAGTTCAGACGTGTGCTCTTCCGATCT
**PCR_reverse_index2**	CAAGCAGAAGACGGCATACGAGATACATCGGTGACTGGAGTTCAGACGTGTGCTCTTCCGATCT
**PCR_reverse_index3**	CAAGCAGAAGACGGCATACGAGATGCCTAAGTGACTGGAGTTCAGACGTGTGCTCTTCCGATCT
**PCR_reverse_index4**	CAAGCAGAAGACGGCATACGAGATTGGTCAGTGACTGGAGTTCAGACGTGTGCTCTTCCGATCT
**PCR_reverse_index5**	CAAGCAGAAGACGGCATACGAGATCACTGTGTGACTGGAGTTCAGACGTGTGCTCTTCCGATCT
**PCR_reverse_index6**	CAAGCAGAAGACGGCATACGAGATATTGGCGTGACTGGAGTTCAGACGTGTGCTCTTCCGATCT
**PCR_reverse_index7**	CAAGCAGAAGACGGCATACGAGATGATCTGGTGACTGGAGTTCAGACGTGTGCTCTTCCGATCT
**PCR_reverse_index8**	CAAGCAGAAGACGGCATACGAGATTCAAGTGTGACTGGAGTTCAGACGTGTGCTCTTCCGATCT
**PCR_reverse_index9**	CAAGCAGAAGACGGCATACGAGATCTGATCGTGACTGGAGTTCAGACGTGTGCTCTTCCGATCT
**PCR_reverse_index10**	CAAGCAGAAGACGGCATACGAGATAAGCTAGTGACTGGAGTTCAGACGTGTGCTCTTCCGATCT
**PCR_reverse_index11**	CAAGCAGAAGACGGCATACGAGATGTAGCCGTGACTGGAGTTCAGACGTGTGCTCTTCCGATCT
**PCR_reverse_index12**	CAAGCAGAAGACGGCATACGAGATTACAAGGTGACTGGAGTTCAGACGTGTGCTCTTCCGATCT
**PCR_reverse_index13**	CAAGCAGAAGACGGCATACGAGATTTGACTGTGACTGGAGTTCAGACGTGTGCTCTTCCGATCT
**PCR_reverse_index14**	CAAGCAGAAGACGGCATACGAGATGGAACTGTGACTGGAGTTCAGACGTGTGCTCTTCCGATCT
**PCR_reverse_index15**	CAAGCAGAAGACGGCATACGAGATTGACATGTGACTGGAGTTCAGACGTGTGCTCTTCCGATCT
**PCR_reverse_index16**	CAAGCAGAAGACGGCATACGAGATGGACGGGTGACTGGAGTTCAGACGTGTGCTCTTCCGATCT
**PCR_reverse_index17**	CAAGCAGAAGACGGCATACGAGATCTCTACGTGACTGGAGTTCAGACGTGTGCTCTTCCGATCT
**PCR_reverse_index18**	CAAGCAGAAGACGGCATACGAGATGCGGACGTGACTGGAGTTCAGACGTGTGCTCTTCCGATCT
**PCR_reverse_index19**	CAAGCAGAAGACGGCATACGAGATTTTCACGTGACTGGAGTTCAGACGTGTGCTCTTCCGATCT
**PCR_reverse_index20**	CAAGCAGAAGACGGCATACGAGATGGCCACGTGACTGGAGTTCAGACGTGTGCTCTTCCGATCT
**PCR_reverse_index21**	CAAGCAGAAGACGGCATACGAGATCGAAACGTGACTGGAGTTCAGACGTGTGCTCTTCCGATCT
**PCR_reverse_index22**	CAAGCAGAAGACGGCATACGAGATCGTACGGTGACTGGAGTTCAGACGTGTGCTCTTCCGATCT
**PCR_reverse_index23**	CAAGCAGAAGACGGCATACGAGATCCACTCGTGACTGGAGTTCAGACGTGTGCTCTTCCGATCT
**PCR_reverse_index24**	CAAGCAGAAGACGGCATACGAGATGCTACCGTGACTGGAGTTCAGACGTGTGCTCTTCCGATCT

Oligonucleotide sequences © 2007-2009 Illumina, Inc. All rights reserved.

Supplementary table 2: Explanation of output from getFreq function

Variable	Description	Example
**chr**	Seqence/chr/scaffold name.	Yeast_LSU_rRNA_U53879
**pos**	Position.	1870
**ref**	Reference base.	C
**totalCounts**	Total number of reads that cover position (including deletions)*.	15675
**relFreq**	Difference in mutation frequency (AltFreqCC1-AltFreqCC0).	0,0083
**CCPval**	P-value for frequency difference between the treated and control samples.	145
**indPval**	P-values of individual samples having mutations.	1;1;14;999;147;999
**CC0Pval**	P-value for differences in mutation frequencies within control samples.	2
**CC1Pval**	P-value for differences in mutation frequencies within treated samples.	12
**SNPPval**	P-value for position being variable.	999
**countsCC0**	Counts of alleles within control samples*.	.=10481,A=1,T=8
**countsCC1**	Counts of alleles within treated sample*.	.=5137,A=2,T=46
**typeCountCC0**	Number of different types of mutation in control samples*.	2
**typeCountCC1**	Number of different types of alleles in treated samples*.	2
**totalCountsCC0**	Total reads covering controls inc. deletions*.	10490
**totalCountsCC1**	Total reads covering treated samples inc. deletions*.	5185
**AltFreq**	Frequency of alt alleles (1-Frequency of ref allele).	0,0034
**AltFreqCC0**	Frequencies of alleles (1-Frequency of ref allele) within control samples.	0,0089
**AltFreqCC1**	Frequencies of alleles (1-Frequency of ref allele) within treated samples.	0,0006
**SNPfreq**	Estimated frequencies (for all samples).	.=0.9966,T=0.0033,A=0
**CC0freq**	Estimated frequencies for control samples.	.=0.9994,T=6e-04,A=0
**CC1freq**	Estimated frequencies for treated samples.	.=0.9911,T=0.0087,A=2e-04
**counts**	Counts of alleles for all samples*.	.=15618,A=3,T=54
**indCounts**	Counts of alleles for each sample*.	.=4915,A=1,T=4;.=4943,T=3;.=623,T=1;.=3092,T=27;.=1010,A=2,T=7;.=1035,T=12
**indFreq**	Estimated allele frequencies for each sample.	.=0.9995,T=5e-04,A=0;.=0.9994,T=6e-04,A=0;.=0.9984,T=0.0016,A=0;.=0.9915,T=0.0085,A=0;.=0.9916,T=0.0068,A=0.0015;.=0.9886,T=0.0114,A=0

* Only alleles that are observed with more than "minCount" in at least one sample are counted. The getFreq function takes the “minCount” as an input and will only perform analysis of the position if the total number of observed mutations at the position (totalCounts) > minCounts.

Supplementary table 3: m7G modification in yeast tRNA

GtRNAdb Gene symbol	Modomics**	Enroth *et. al*	Marchand *et. al*
**Yeast_tRNA-Ala-AGC-1**	-	-	+
**Yeast_tRNA-Cys-GCA-1**	+	+*	+
**Yeast_tRNA-Ile-TAT-1**	+	+	
**Yeast_tRNA-Ile-TAT-2**	+	+	
**Yeast_tRNA-iMet-CAT-1**	+	+	+
**Yeast_tRNA-Lys-CTT-1**	+	+	+
**Yeast_tRNA-Lys-TTT-1**	+	+*	+
**Yeast_tRNA-Met-CAT-1**	+	+*	+
**Yeast_tRNA-Phe-GAA-1**	+	+	
**Yeast_tRNA-Phe-GAA-2**	+	+	+
**Yeast_tRNA-Pro-AGG-1**		+	
**Yeast_tRNA-Pro-TGG**	+	+	+
**Yeast_tRNA-Thr-TGT-2**		+	
**Yeast_tRNA-Trp-CCA-1**	+	+	+
**Yeast_tRNA-Val-CAC-1**	+	+	+
**Yeast_tRNA-Val-AAC-1**	+	+	+

*The three tRNAs, met-CAU, Lys-UUU and Cys-GCA all have increased relative frequency and -10*log transformed p-value, but does not reach our cut-off, most likely due to low coverage. Conversely, these tRNA obtain the highest normalized cleavage values in Marchand et. al, which may indicate that abasic sites created in these tRNA are more prone to strand breakage.
** MODOMICS: a database of RNA modification pathways (5).

Supplementary references

1. Li, H. (2011) Improving SNP discovery by base alignment quality. *Bioinformatics*, **27**, 1157-1158.
2. Li, H. (2011) A statistical framework for SNP calling, mutation discovery, association mapping and population genetical parameter estimation from sequencing data. *Bioinformatics*, **27**, 2987-2993.
3. Kielpinski, L.J., Sidiropoulos, N. and Vinther, J. (2015) In Sarah, A. W. and Frédéric, H. T. A. (eds.), *Methods Enzymol*. Academic Press, Vol. Volume 558, pp. 153-180.
4. McLuckey, S.A., Van Berkel, G.J. and Glish, G.L. (1992) Tandem mass spectrometry of small, multiply charged oligonucleotides. *J Am Soc Mass Spectrom*, **3**, 60-70.
5. Machnicka, M.A., Milanowska, K., Osman Oglou, O., Purta, E., Kurkowska, M., Olchowik, A., Januszewski, W., Kalinowski, S., Dunin-Horkawicz, S., Rother, K.M. *et al.* (2013) MODOMICS: a database of RNA modification pathways--2013 update. *Nucleic Acids Res*, **41**, D262-267.
